# Ferroelectric Optoelectronic Sensor for Intelligent Flame Detection and In-Sensor Motion Perception

**DOI:** 10.1007/s40820-025-01968-x

**Published:** 2026-01-13

**Authors:** Jiayun Wei, Guokun Ma, Runzhi Liang, Wenxiao Wang, Jiewei Chen, Shuang Guan, Jiaxing Jiang, Ximo Zhu, Qian Cheng, Yang Shen, Qinghai Xia, Shiwen Wu, Houzhao Wan, Longhui Zeng, Mengjiao Li, Yi Wang, Liangping Shen, Wei Han, Hao Wang

**Affiliations:** 1 School of Integrated Circuits, Hubei University, Wuhan, 430062 People’s Republic of China; 2 School of Physics, South China Normal University, Guangzhou, 510006 People’s Republic of China; 3 School of Physics and Microelectronics, Key Laboratory of Material Physics Ministry of Education, Zhengzhou University, Zhengzhou, 450052 People’s Republic of China; 4 School of Microelectronics, Shanghai University, Jiading, Shanghai 201800 People’s Republic of China; 5 Center for Microscopy and Analysis, Nanjing University of Aeronautics and Astronautics, Nanjing, 210016 People’s Republic of China

**Keywords:** Gallium oxide, Indium selenide, Flame detection, Flame motion recognition

## Abstract

Next-generation fire safety systems demand precise detection and motion recognition of flames. In-sensor computing, which integrates sensing, memory, and processing capabilities, has emerged as a key technology in flame detection. However, the implementation of hardware-level functional demonstrations based on artificial vision systems in the solar-blind ultraviolet (UV) band (200–280 nm) is hindered by the weak detection capability. Here, we propose Ga_2_O_3_/In_2_Se_3_ heterojunctions for the ferroelectric (abbreviation: Fe) optoelectronic sensor (abbreviation: OES) array (5 × 5 pixels), which is capable of ultraweak UV light detection with an ultrahigh detectivity through ferroelectric regulation and features in configurable multimode functionality. The Fe-OES array can directly sense different flame motions and simulate the non-spiking gradient neurons of insect visual system. Moreover, the flame signal can be effectively amplified in combination with leaky integration-and-fire neuron hardware. Using this Fe-OES system and neuromorphic hardware, we successfully demonstrate three flame processing tasks: achieving efficient flame detection across all time periods with terminal and cloud-based alarms; flame motion recognition with a lightweight convolutional neural network achieving 96.47% accuracy; and flame light recognition with 90.51% accuracy by means of a photosensitive artificial neural system. This work provides effective tools and approaches for addressing a variety of complex flame detection tasks.

**Supplementary Information:**

The online version contains supplementary material available at 10.1007/s40820-025-01968-x.

## Highlights

- The Ga₂O₃/In₂Se₃ heterojunction ferroelectric optoelectronic sensor array enables precise detection of ultraweak UV signals through ferroelectric modulation.
- Efficient flame detection across all time periods is achieved through terminal devices and cloud-based alert systems.
- The lightweight convolutional neural network-based approach achieves a flame motion recognition accuracy of 96.47%, while the optoelectronic artificial neural system attains 90.51% accuracy in identifying flame optical signals.

**Supplementary Information:**

The online version contains supplementary material available at 10.1007/s40820-025-01968-x.

## Graphical abstract

**Figure:**
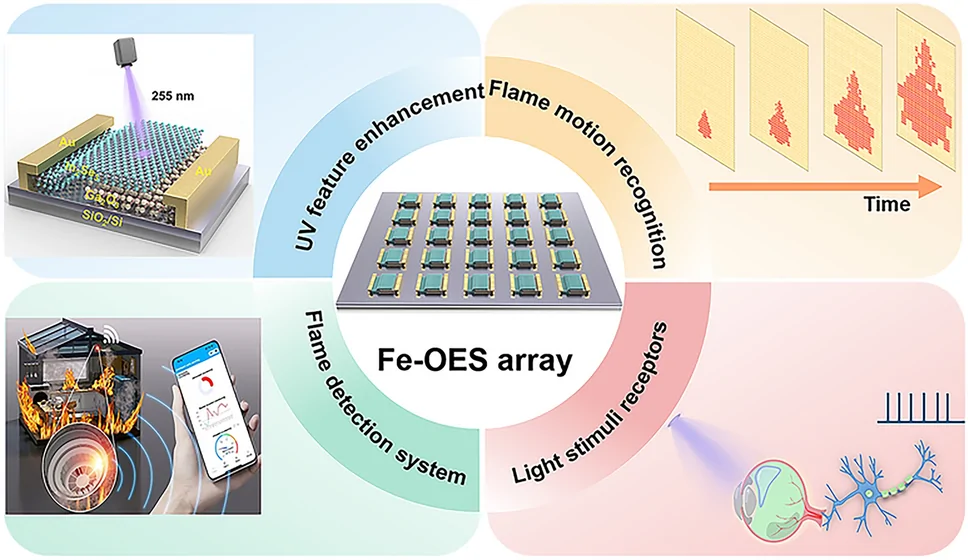

## Introduction

Fire accidents cause significant loss of life and property globally each year [1, 2]. Traditional flame detection systems, which rely on smoke and infrared heat sensors activated by combustion, are limited to issuing basic flame alarms or capturing spatial frames [3, 4]. These limitations make it challenging to accurately assess the intensity and spread of the fire [5, 6]. It is noted that the solar-blind UV (SBUV, 200–280 nm) rays can emit rapidly within 3 to 4 ms during material combustion, which stimulates the further research to explore the ultraviolet flame detectors with high sensitivity and fast response. To enhance fire prevention capabilities, the development of an advanced vision system is critically need, which integrates real-time monitoring of SBUV components with spatial and temporal analysis algorithms to enable early-stage flame detection and dynamic fire behavior assessment [7, 8]. Vision systems for motion recognition often employ various artificial neural network algorithms, such as convolutional neural networks (CNN) [9–11] and spiking neural networks (SNN) [12–14], to reduce the transmission of large volumes of visual data while achieving spatiotemporal integration within computational units [15–17]. Consequently, systems that integrate sensors and external circuits to generate, collect, and process flame data present a viable approach [18, 19], significantly improving data processing efficiency and minimizing redundant transmission [20–23]. The detection with artificial vision systems in visible to infrared light regions has been extensively investigated, while SBUV-based artificial vision for weak-light detection remain underdeveloped with limited hardware-level demonstrations, presenting a critical gap in flame detection technology.

As a representative SBUV detection material, ultra-wide bandgap Ga_2_O_3_ shows strong recognition and anti-interference capabilities for flame detection and missile warning [24, 25]. Nevertheless, the insufficiency of weak-light detection (nW cm^−2^) in Ga_2_O_3_-based detectors still hinders their commercialization [26]. Ferroelectric polarization engineering can break this dilemma via voltage-driven spontaneous polarization alignment. It is worth noting that the ferroelectric negative capacitance effect can be generated when ferroelectric material is used as gate medium of field-effect transistor (FET). In this case, the ferroelectric polarization field enables the suppression of dark current and enhancement of photodetection capability, which finally optimize the detectivity under weak illumination conditions [27]. This improvement has been widely observed in two-dimensional (2D) devices, such as HZO-gated-MoS_2_ phototransistor [27] and P(VDF-TrFE)-gated-MoTe_2_ p–n junction [28]. As a 2D van der Waals ferroelectric semiconductor, α-In_2_Se_3_ exhibits in-plane and out-of-plane ferroelectricity at room temperature, along with excellent ferroelectric control [29–33]. By applying a scanning voltage, the ferroelectric polarization in α-In_2_Se_3_ can be effectively switched, enabling the ferroelectric local field effect to selectively modulate background charge carriers [34]. This field adjustment effectively captures more holes to enhance the optical gating effect and amplifies the ferroelectric negative capacitance effect, achieving ultrahigh photodetection rates while maintaining high responsivity and detectivity [35, 36].

Here, we present a 5 × 5 ferroelectric (abbreviation: Fe) optoelectronic sensor (abbreviation: OES) array based on Ga_2_O_3_/In_2_Se_3_ heterojunctions. With its excellent device quality, the Fe-OES exhibits outstanding photoelectric response under 255 nm solar-blind UV light, enabling the detection of extremely weak flames and efficiently encoding spatiotemporal information at the sensing terminal. Furthermore, with external circuit integration and sensor-intrinsic computation, we demonstrate three representative flame detection applications using the Fe-OES biomimetic vision system: low-level feature-enhanced flame detection experiments, high-level flame motion recognition simulations, and advanced hardware experiments for biomimetic flame perception. Fe-OES features manufacturing compatibility with external circuits and adjustable multimode functionalities, supporting various neural networks (e.g., CNN and SNN), thus offering a versatile platform for artificial vision-based flame detection systems.

## Experimental Section

### Preparation of Ga_2_O_3,_ and In_2_Se_3_

#### ***Growth of Ga***_***2***_***O***_***3***_*** Films***

The synthesis of Ga_2_O_3_ thin films was achieved through atomic layer deposition (ALD). The specific process involved the introduction of a clean and dry Si substrate, coated with a 300 nm SiO_2_ insulating layer, into the chamber of the ALD equipment. The substrate was subjected to oxygen plasma for a period to precondition the surface and remove contaminants from the substrate surface. Trimethylgallium was deposited for 0.1 s, followed by 20 s of argon purging, and then 10 s of oxygen exposure. The typical growth temperature is about 300 °C.

#### ***Growth of In***_***2***_***Se***_***3***_*** Films***

Approximately 100 mg of In_2_Se_3_ powder was placed in a quartz boat located at zone 2 of the heating area, evenly spread out. Commercial mica (KMg_3_AlSi_3_O_10_F_2_) sheets were used as substrates, placed directly above the In_2_Se_3_ powder, and the vertical distance between the source material and the substrate was controlled within a narrow range (approximately 3 mm). Prior to the reaction, the system was evacuated under preliminary vacuum, followed by maintaining a vacuum level of approximately 0.5 Pa. The target temperature of the heating zone was typically set to 800 °C. After reaching the target temperature, the holding time was typically 30 min. The heating rate was typically 33 °C min^−1^. After the reaction, the quartz boat was slowly pulled out of the heating zone using a magnet, and the product cooled down to room temperature in the furnace, resulting in a 2D In_2_Se_3_ film on the mica substrate.

#### ***Transfer Method of In***_***2***_***Se***_***3***_*** Films***

First, the surface of mica sheets grown with two-dimensional phase In_2_Se_3_ films was coated with a layer of PMMA solution using a spin coater at 2000 rpm. Subsequently, the PMMA-coated samples were baked on a hot plate at 80 °C for 5 min to evaporate the organic solvent in the PMMA, thereby solidifying it into a film. The samples were then surrounded with tape, immersed in deionized water, and using tweezers, the PMMA film containing the sample was peeled off from the mica due to the surface tension of the water. Next, the PMMA film with the sample was transferred onto a Ga_2_O_3_ film and baked at 45 °C on a hot plate for 10 min to completely evaporate the water. After dissolving the PMMA with acetone solution, the In_2_Se_3_ film was transferred onto the Ga_2_O_3_ film.

### Materials Characterizations

The In_2_Se_3_/Ga_2_O_3_ heterojunction was characterized by OM (Ningbo Sunny, CX40M), KPFM and PFM (Oxford Instruments Asylum Research and Cypher S), and XPS (Thermo Fisher Scientific K-Alpha); all samples were analyzed using an Al Ka X-ray source (spot size of 400 μm) at a constant dwelling time for 100 ms wide scan (single scan, a step size of 1 eV) and 300 ms narrow scan (five scans, a step size of 0.05 eV). The survey spectra and high-resolution single core-level spectra were measured at the pass energies of 150 and 30 eV, respectively. To neutralize the charge on the sample during the experiments, an electron–ion charge compensation system was used. The studies were carried out under ultrahigh vacuum 10^–9^ mba at room temperature; in the case of using a sample charge compensation system, the partial pressure of argon in the analytical chamber was 5 × 10^–7^ mbar. The experimental data were processed using Thermo Fisher Scientific K-Alpha, the spectrometer software (Avantage Thermo Fisher Scientific). The cross-sectional sample was made by focused-ion beam (FIB, Thermo Fisher Helios5 UX), and the TEM/EDS data were collected by a spherical aberration-corrected TEM (JEOL JEM-ARM200F).

### Device Fabrication and Characterization

Au electrodes were deposited by a thermal evaporation coater by using TEM grids (Zhongjingkeyi) as hard masks. Following the removal of the grids, the Au electrodes were fabricated. All current–voltage (*I–V*) and current–time (*I–t*) curves were measured in a vacuum environment in the probe station (MyProber) using the semiconductor analyzer (Keithley 4200-SCS). The noise current is measured by PDA PXle-FS380 and Keithley 4200A-SCS. The light source used for the measurement was a LED 255 nm whose optical power was measured by a standard silicon photodiode and adjusted by a neutral density optical filter.

### Flame Detection Alarm System

The system comprises an In_2_Se_3_/Ga_2_O_3_ heterojunction flame detector, a smoke detector, a detector scanning platform, a detector *I–V* amplifier circuit, an ESP-Wroom-32 master control circuit, and an NB-IoT communication circuit. The specific operation principle is as follows: When a flame is detected, the flame detector senses it, causing a change in the detector’s current. This changed current then passes through the *I–V* amplifier circuit. The ESP-Wroom-32 master control unit reads the amplified voltage signal and subsequently transmits the information via WiFi or the NB-IoT network.

## Results and Discussion

### Characterization and Performance of Fe-OES

**Fig. 1:**
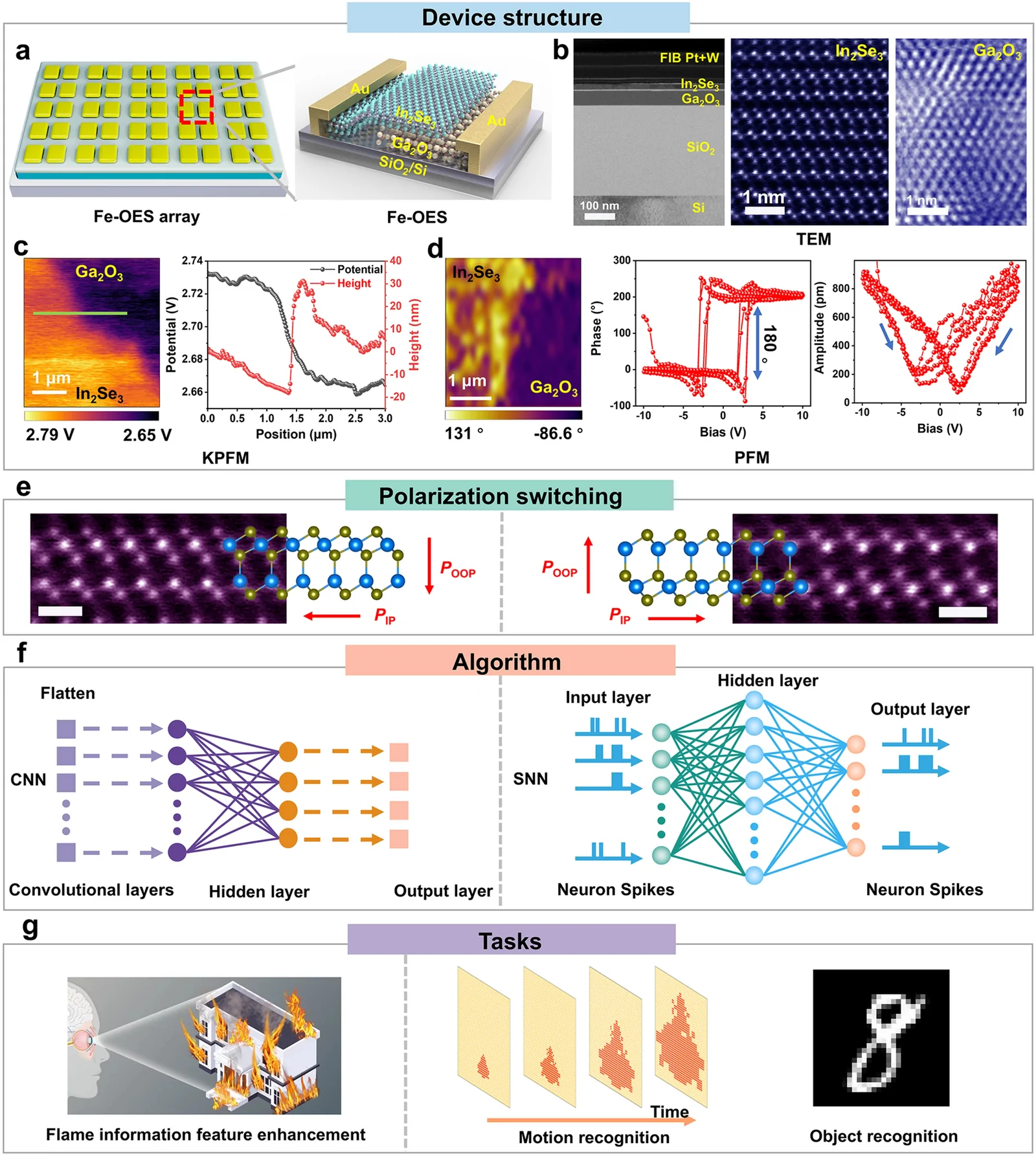
Fe-OES system. **a** Schematic structure of the 5 × 5 array. **b** Cross-sectional TEM and HAADF-STEM images at the Ga_2_O_3_/In_2_Se_3_ interface. The thickness of α-In_2_Se_3_ and Ga_2_O_3_ is 15 nm and 60 nm, respectively. **c** KPFM image of the Ga_2_O_3_/In_2_Se_3_ interface, including height and potential differences. **d** (left) PFM phase mapping of the Ga_2_O_3_/In_2_Se_3_ interface. (middle) Hysteresis loop with a phase difference of 180°. (right) The corresponding amplitude hysteresis loop. **e** Atomic STEM images and the corresponding crystal structures of α-In_2_Se_3_ with down/up polarized states (out of plane) and left/right polarized states (in-plane). Scale bar, 0.5 nm. The yellow and blue balls represent Se and In atoms, respectively. **f** Diagram of CNN and SNN algorithms supported through Fe-OES. **g** Illustration of a multi-scenario demonstration of flame detection, flame motion recognition, and flame biosensing with the Fe-OES system

The large-area growth of Ga_2_O_3_ and In_2_Se_3_ has enabled the fabrication of device arrays based on Ga_2_O_3_/In_2_Se_3_ heterojunction. Specifically, Wafer-scale Ga_2_O_3_ thin films were prepared by atomic layer deposition (ALD) and the centimeter-scale *α*-In_2_Se_3_ single-crystal was synthesized by chemical vapor deposition (CVD). The experimental details can be found in methods. Photographs of the Ga_2_O_3_ and In_2_Se_3_ thin films are shown in Fig. S1. The schematic structure of the Ga_2_O_3_/In_2_Se_3_ Fe-OES array are shown in Fig. 1a. The cross-sectional transmission electron microscopy (TEM) image in Fig. 1b exhibits clear boundaries between each layer, and its corresponding elemental mapping images of the Ga_2_O_3_/In_2_Se_3_ interface (Fig. S2) further confirm the uniform distribution of In, Se, O, and Ga elements. The single-crystal layered structure of In_2_Se_3_ and the polycrystalline nature of Ga_2_O_3_ were verified by the high-angle annular dark-field scanning transmission electron microscopy (HAADF-STEM) in Fig. 1b.

Based on the Kelvin probe force microscopy (KPFM) measurement of the Ga_2_O_3_/In_2_Se_3_ heterojunction in Fig. 1c, the potential difference between them can be detected as ~ 70 mV, effectively confirming the formation of a built-in potential at the junction interface. Additionally, a direct voltage sweep from -10 to + 10 V was applied to the Ga_2_O_3_/ In_2_Se_3_ heterojunction, revealing a phase hysteresis loop with a 180° phase difference and the amplitude hysteresis loop. This confirms the out-of-plane ferroelectricity of the Ga_2_O_3_/In_2_Se_3_ heterojunction, as observed through piezoelectric force microscopy (PFM) (Fig. 1d). As shown in Fig. 1e, atomic STEM images and the corresponding crystal structures of α-In_2_Se_3_ reveal the down/up polarized states (out of plane, OOP) and left/right polarized states (in-plane, IP). The concurrent reversal of both OOP and IP polarization components originates from the coordinated displacement of central selenium atoms [31].

As shown in Fig. 1f, the multi-modal responses of Fe-OES can support various application scenarios and algorithms, such as CNNs and SNNs. We propose diversified flame detection application scenarios based on Fe-OES (Fig. 1g). These include low-level processing experiments for feature-enhanced flame detection, high-level simulations for flame motion recognition, and advanced hardware experiments for biomimetic flame perception in artificial visual neurons.

The X-ray photoelectron spectroscopy (XPS) of Ga_2_O_3_ and In_2_Se_3_ was performed to explore the crystal quality. The binding energies of Ga 2*p*_3/2_, Ga 2*p*_1/2_, and Ga 3*d* for unannealed and annealed Ga_2_O_3_ films are presented in Fig. S3a–c. Shifts in the Ga 2*p* and Ga 3*d* spectra toward lower energies indicate the presence of abundant Ga-Ga units in the Ga_2_O_3_ films [37]. For In_2_Se_3_, Fig. S3d–f illustrates the In 3*d*, Se 3*d*, and Se 3*p* peaks of In_2_Se_3_ films, thereby corroborating the successful growth of In_2_Se_3_ films. To further obtain the optimal device performance, we systemically compared Ga_2_O_3_ films prepared without annealing, annealed at 400 °C in air, and annealed at 700 °C in air. Figure S4 presents a representative device based Ga_2_O_3_ film with the two-electrode structure (electrode width: ~ 108 μm, a channel length: 37 μm, and an effective device area ~ 39.96 μm^2^). As a matter of fact, all optoelectronic characterizations demonstrate that the device annealed at 400 °C exhibits superior detection performance compared to its counterparts (Fig. S5). Figures S6–S8 illustrate the photoelectric performance of non-annealed, 400 °C-annealed, and 700 °C-annealed Ga_2_O_3_ devices, respectively. The formulas of responsivity (*R*) and detectivity (D*) are as follows [38]:

1 $$\begin{array}{*{20}c} {{\text{R = }}\frac{{{\mathrm{I}}_{{{\mathrm{light}}}} {\text{ - I}}_{{{\mathrm{dark}}}} }}{{{\text{S P}}_{{{\mathrm{light}}}} }}} \\ \end{array}$$

2 $$\begin{array}{*{20}c} {{\text{D* = }}\frac{{{\text{R }}\sqrt {\text{S f}} }}{{{\mathrm{I}}_{{{\mathrm{noise}}}} }}} \\ \end{array}$$

where *S* refers to the irradiated working area of device, *P* is the optical power density, *I*_light_ is the photocurrent, *I*_dark_ is the dark current, *f* is the detection line width, and *I*_noise_ is the noise current.

As illustrated in Figs. S7a and S8a, the dark current of 400 °C-annealed Ga_2_O_3_ device decreases from 0.15 to 0.11 nA. The 700 °C-annealed device exhibits an ultra-low dark current of only 0.41 pA at 5 V bias, but with a photocurrent of only 0.1 nA at maximum light intensity. Figures S7b and S8b show the noise currents of the two devices, which are 1.23 × 10^–14^ and 1.26 × 10^–14^ A/Hz^−1/2^ at 100 Hz frequency, respectively. Figure S7c shows that at 1 V bias, the *R* and D^*^ of the 400 °C-annealed Ga_2_O_3_ devices are 486 A W^−1^ and 2.45 × 10^15^ Jones, both higher than those of the non-annealed and 700 °C-annealed devices. Furthermore, the external quantum efficiency (*EQE*) represents the ratio of the number of charge excitons collected in SBPD to the number of incidents lights [39], the detailed results are shown in Figs. S6d, S7d, and S8d. Ga₂O₃ photodetectors exhibit persistent photoconductivity (PPC) effect [27], a phenomenon where the conductivity of the detector persists long after the cessation of illumination. This phenomenon is primarily attributed to deep-level defects, such as oxygen vacancies (V_O_), which trap photogenerated carriers can enhance non-radiative recombination and prolong the conductive state. Furthermore, these defects contribute to higher dark current and low specific detectivity. Conversely, V_O_ can also temporarily trap electrons under illumination, influencing the photocurrent generation and consequently degrading the response speed. Therefore, modifying the intrinsic crystalline defects in Ga₂O₃ is crucial for enhancing device performance. Annealing engineering serves as a common strategy for this purpose. The specific effects of annealing treatments on Ga₂O₃ photodetectors, as reported in the literature, are summarized in Table S1. High-temperature annealing can effectively suppress intrinsic defects in Ga₂O₃, leading to devices with lower dark current and higher responsivity. This explains the significant performance improvement observed in the device annealed at 400 °C. However, although annealing at even higher temperatures (e.g., 700 °C) leads to more pronounced defect suppression, it also results in a substantial reduction in photocurrent. This is because V_O_, while suppressed, can still act as temporary electron traps under illumination, adversely affecting the overall photoelectronic performance. This mechanism accounts for the performance degradation observed in the sample annealed at 700 °C compared to the one annealed at 400 °C.

**Fig. 2:**
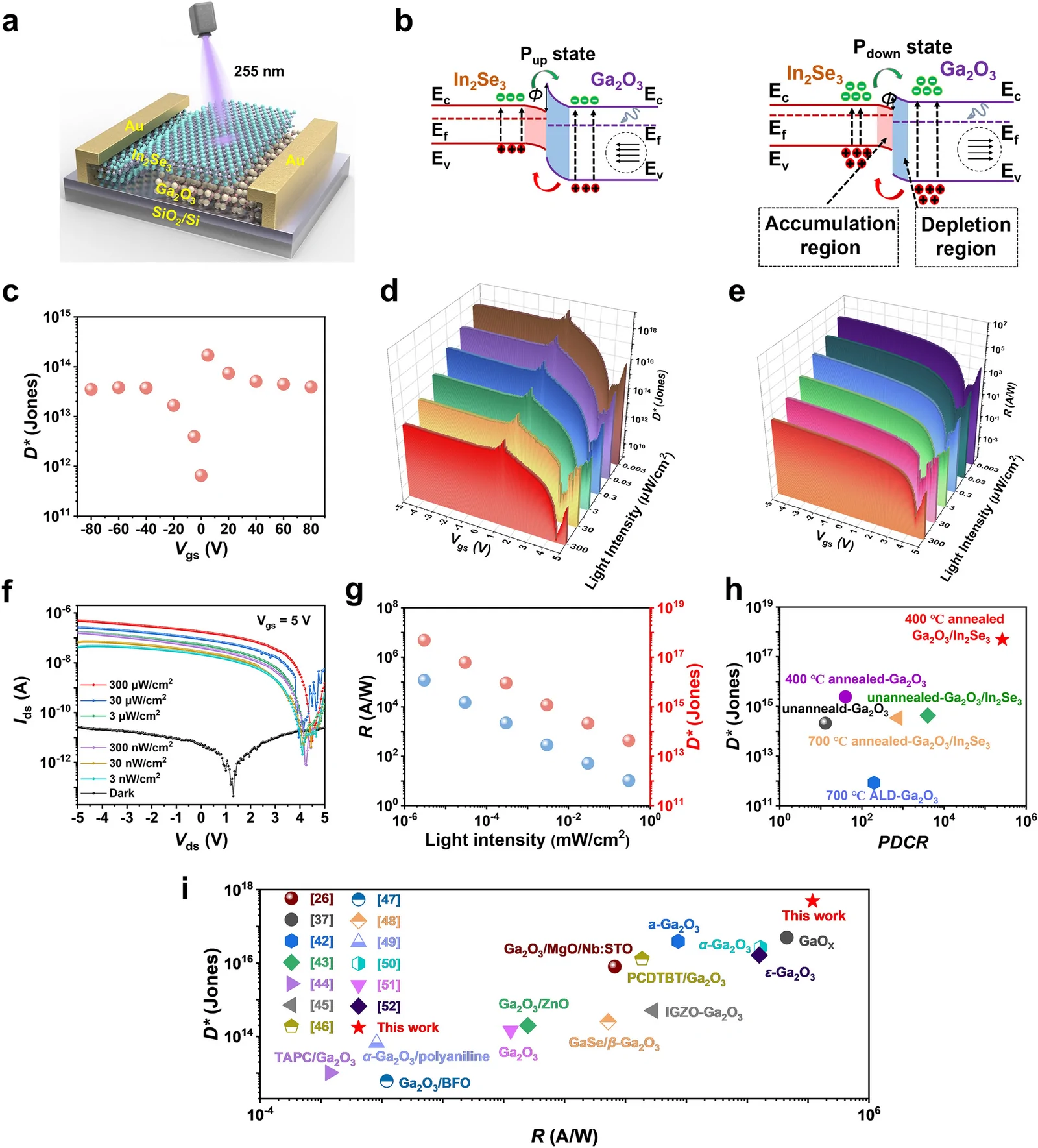
Photoelectric performance of Fe-OES. **a** Schematic of the Fe-OES device. **b** The energy band alignments of Ga_2_O_3_/In_2_Se_3_ heterojunction under P_down_ and P_up_ state. The double arrow line represents the barrier height (*ϕ*). The circular dashed area indicates the polarization direction of the In_2_Se_3_. **c **D* curves for different gate voltages in 300 μW/cm^2^ of 255 nm. **d, e** D* and *R* data under different gate voltages and light intensities. **f** *I*_ds_–*V*_ds_ image for different light intensities. **g** *R* and D* at 5 V gate voltage. **h** D* and *PDCR* comparison of Fe-OES and other Ga_2_O_3_-based devices. **i** Comparison of *R* and D* with other references

Figure 2a schematically illustrates Ga_2_O_3_/In_2_Se_3_ heterojunction devices, and the energy band alignments of heterojunction under *P*_down_ and *P*_up_ state are shown in Fig. 2b. In the polarization downward state, the polarization direction of the In_2_Se_3_ is from the In_2_Se_3_ to Ga_2_O_3_. In the polarization upward state, the polarization direction of the In_2_Se_3_ is from the Ga_2_O_3_ to In_2_Se_3_. Figure S9a shows the In_2_Se_3_ film before transfer, while the step-wise fabrication process of Ga_2_O_3_/In_2_Se_3_ heterojunction device is shown in Fig. S9b. Detailed photoelectric performance testing was conducted on the Ga_2_O_3_/In_2_Se_3_ heterojunction device prepared using Ga_2_O_3_ film annealed at optimal temperature (~ 400 °C), as shown in Fig. S10a. The device exhibits obvious responsiveness to 30 nW cm^−2^ intensity under 255 nm light illuminating. The device’s dark current is 13.7 pA at 1 V bias, which is approximately one order of magnitude lower than that of the Ga_2_O_3_ device annealed at 400 °C. Moreover, to gain insight into the particular enhancements of the heterojunction device, calculations were performed for additional photoelectric characteristics, including *I*_*noise*_, *R*, D*, and *EQE*, as illustrated in Fig. S11b–d. The *R* increases from 486 to 21,244 A W^−1^, the D* increases from 2.45 × 10^15^ to 1.08 × 10^17^ Jones, and the *EQE* increases from 236,789% to 10,294,805%, exhibiting a good photoelectric response even at lower light intensities.

To further validate the performance enhancement of the heterojunction device compared to Ga_2_O_3_-based control devices, heterojunction devices were also fabricated with the non-annealed Ga_2_O_3_ film and the annealed Ga_2_O_3_ film at 700 °C. For the non-annealed Ga_2_O_3_ film, as shown in Fig. S11, *R* of the heterojunction device increases from 116 A/W to 236 A W^−1^, D* increases from 2.11 × 10^14^ to 4.33 × 10^15^ Jones, and *EQE* increases from 56,498% to 115,040%. For Ga_2_O_3_ thin films annealed at 700 °C in Fig. S12, the heterojunction devices exhibited substantial improvements. *R* increased dramatically from 0.28 to 104 A W^−1^. Similarly, the D* demonstrated a significant enhancement, rising from 8.51 × 10^11^ to 3.44 × 10^14^ Jones. Furthermore, the *EQE* exhibited substantial growth, increasing from 138% to 50,822%. These enhancements underscore the transformative potential of heterojunction engineering in advancing optoelectronic performance and paving the way for high-performance UV photodetectors.

The observed reduction in the dark current of the heterojunction device can be attributed to the potential barrier at the heterointerface in Fig. S13. In the *P*_down_ state, due to the lower built-in electric field strength/barrier height, a greater number of carriers can pass through the region of the built-in electric field, resulting in a larger current than in the pristine state. Conversely, in the *P*_up_ state, the higher built-in electric field strength/barrier height impedes the transfer of charge carriers, resulting in a smaller current. The negative capacitance effect enhances the built-in electric field, leading to a significant increase in the Schottky barrier or carrier injection barrier at the metal–semiconductor interface, as illustrated in Fig. S13a, b. This suppresses electron injection from the metal into the conduction band, thereby reducing the dark current caused by thermal excitation [27, 36]. On the other hand, it blocks leakage current paths, mitigating carrier diffusion or tunneling under dark conditions, which further contributes to the suppression of dark current. Moreover, due to the amplification of the internal electric field strength per unit applied voltage by negative capacitance (Fig. S13c, d), particularly within the depletion region or at heterojunction interfaces, the strengthened built-in field promotes more efficient separation of photogenerated electron–hole pairs. This reduces the recombination rate and enhances the collection efficiency of minority carriers [34, 36]. Furthermore, under weak incident light, the photocurrent induced by low-intensity illumination can be collected and amplified more rapidly and effectively owing to the negative capacitance effect. This enables the device to achieve high photodetection performance even under extremely low-light conditions.

Electrical characterization of the Ga₂O₃/In₂Se₃ heterojunction was performed. As shown in Fig. S14, the transfer characteristics of the heterojunction device under various drain–source voltages (*V*_ds_) reveal a clockwise hysteresis window exceeding 40 V during gate voltage sweeps from -40 to 40 V. As *V*_ds_ increases, both the on/off ratio and the hysteresis window expand further, demonstrating the strong potential of the heterojunction device for in-sensor computing applications. *I*_*ds*_–*V*_*ds*_ tests were conducted on heterojunction devices prepared with Ga_2_O_3_ films that had undergone annealing at 400 °C, at varying gate voltages. These results, presented in Fig. S15, reveal a strong dependence of the dark and photocurrents on the polarization state, particularly influenced by the ferroelectric polarization of the In_2_Se_3_. Subsequently, specific optoelectronic data under different gate voltages at *V*_*ds*_ = 1 V were extracted and calculated, resulting in the D* plots under different gate voltages shown in Fig. 2c. The analysis indicates that the device exhibits optimal detectivity at *V*_*gs*_ = 5 V. Figure 2d, e illustrates the relationship between the *R* and D* under different light intensities and bias voltages, respectively. The results demonstrate that both *R* and D* increase as the light intensity decreases. This phenomenon may be attributed to the increase in the concentration of free electrons with increase in incident light power, which enhances carrier scattering. Nevertheless, the rise in free electrons gives rise to an increase in electron–hole recombination, which in turn results in a decline in *R* and D* with rising light intensity.

Subsequent *I*_*ds*_*–V*_*ds*_ testing was conducted on heterojunction devices with a *V*_*gs*_ of 5 V under different light intensities, as illustrated in Fig. 2f. By controlling the gate voltage, the dark current was further suppressed from 13.7 to 0.47 pA at a fixed *V*_*ds*_ = 1 V. However, there was no significant suppression in response to the blind UV signal. The application of a gate voltage enabled the device to demonstrate enhanced capabilities in the detection of blind UV signals. Figure 2g demonstrates the specific optoelectronic performance extracted and analyzed from Fig. 2f. The noise current of the Ga_2_O_3_/In_2_Se_3_ heterojunction device at 5 V gate voltage is shown in Fig. S16. The device noise current reaches a lower value of 1.53 × 10^–14^ A Hz^−1/2^ at 100 Hz frequency, which represents a key factor for its excellent detection performance. Under 3 nW cm^−2^ of 255 nm incident light, Fe-OES devices exhibit high responsivity up to 1.18 × 10^6^ A W^−1^, high detectivity of 4.91 × 10^17^ Jones, and excellent *EQE* of 57,721,079% under *V*_*gs*_ = 5 V and *V*_*ds*_ = 1 V.

In structures with Ohmic or Schottky contacts, the overall resistance is governed by the bulk resistance, and their behavior can be described using a photoconductor model. The gain *G* in such structures is typically explained by carrier recirculation mechanisms. This model assumes that the photocurrent originates from a reduction in resistance due to an increase in carrier concentration. Under illumination, if the excess concentrations of holes and electrons are equal (n = p), the gain can be expressed as [40, 41]:

3 $$\begin{array}{*{20}c} {{\text{G = }}\frac{{{\uptau } \mu_{p} E}}{{\mathrm{l}}}\left( {{1 + }\frac{{{\upmu }_{{\mathrm{n}}} }}{{{\upmu }_{{\mathrm{p}}} }}} \right){ = }\frac{{\uptau }}{{{\mathrm{t}}_{{{\mathrm{tr}}}} }}\left( {{1 + }\frac{{{\upmu }_{{\mathrm{n}}} }}{{{\upmu }_{{\mathrm{p}}} }}} \right)} \\ \end{array}$$

where τ denotes the excess carrier lifetime (assumed equal for electrons and holes), µ_n_ and µ_p_ represent the electron and hole mobilities, respectively, *l* is the length of the illuminated region, *E* is the electric field, and *t*_tr_ is the transit time of minority carriers under a uniform electric field. The record-high detectivity observed in Ga₂O₃ appears to originate, at least in part, from hole trapping within the MSM structure. This trapping mechanism contributes significantly to the photoconductive gain by prolonging the lifetime of photogenerated electrons. The dominant defects responsible for this hole trapping are likely various types of gallium vacancy acceptors. A key trade-off in this unconventional photoconduction mode is the need to dissipate the charge accumulated by holes in deep traps after illumination ceases. In most cases, both the rise and decay of the photocurrent are relatively slow, resulting in a limited device response speed. Therefore, improving the response speed represents a critical direction for our future research. In addition, the crystalline quality of Ga₂O₃ thin films grown on Si substrates is considerably lower than that of high-quality Ga₂O₃ layers epitaxially grown on native substrates, and typically contains multiple structural defects that contribute to additional charge trapping. These defects, including surface and interfacial states, lead to the trapping of majority carriers and result in the formation of a potential barrier and a corresponding depletion region near the surface. The spatial separation of photogenerated electrons and holes suppresses their recombination and extends the effective carrier lifetime, thereby enhancing the overall device gain. The high external quantum efficiency (*EQE*) observed in these devices can be attributed to the significantly elevated τ/t_tr_ ratio.

The comparative performance of the devices is presented in Fig. 2h, i. Figure 2h highlights the performance differences among devices annealed at three self-determined temperatures and their corresponding heterojunctions. According to the comparison between optimal heterojunction device and other reported work (Table S2 [26, 37, 42–52]) in Fig. 2i, Ga_2_O_3_/In_2_Se_3_ heterojunction achieves one of the highest performances among Ga_2_O_3_-based solar-blind UV photodetectors. Additionally, the device performs excellent electrical stability under prolonged optical stimulation. As shown in Fig. S17a, b, the current value retains 95% of its initial performance after 1200 s continuous illumination, and maintains 80% after 12 h continuous illumination. Furthermore, the device has a fast response time of 59 ms as depicted in Fig. S17c. The excellent performance of the device can be largely attributed to the polarized state of In₂Se₃. Stability tests were conducted under a gate voltage of 20 V (Fig. S18a), showing a device performance retention of 89% after 10,000 s of continuous operation. Additionally, cyclic gate voltage tests between 20 and –20 V were performed (Fig. S18b), resulting in a performance retention of 94% after 10,000 s. These results indicate that the device exhibits excellent stability under both prolonged operation and repeated switching cycles. Long-term performance under harsh conditions—such as high humidity and temperature fluctuations—is critical for fire safety applications. To evaluate this, stability tests were conducted over 3000 s under high humidity and varying temperatures, as shown in Fig. S19. The device retained 75% of its initial performance after 3,000 s at 28.2 °C and 66% relative humidity, and 79% at 22.4 °C and 60% relative humidity. The exceptional SBUV photoelectric response of our devices offers the potential for the development of practical applications in flame detection.

The device uniformity across the entire area is important. To evaluate the overall uniformity of the photoelectronic properties across the Ga₂O₃ film, five distinct regions on a 4-inch wafer were selected, and their photocurrents and dark currents were measured. As shown in Fig. S20a, the specific values of the photocurrent and dark current at 5 V are summarized in Fig. S20b. Both the photocurrent and dark current exhibit good uniformity, which is attributed to the high homogeneity of Ga₂O₃ film grown by ALD. Regarding the overall photoelectronic performance of the In₂Se₃ film, five different regions of the Ga₂O₃/In₂Se₃ heterostructure were randomly selected (Fig. S20c, d). The ferroelectric properties were characterized by conducting FET measurements on the In₂Se₃ film at these five regions. The corresponding transfer characteristic curves are shown in Fig. S21, revealing variations in the on/off current ratio and hysteresis window among the five devices.

The variations in both photoelectronic and ferroelectric properties are closely related to the thickness, crystallinity, and polarization of the In₂Se₃ film. Based on this, we investigated In₂Se₃ films with different thicknesses. Figure S22a shows an In₂Se₃ film grown on mica prior to transfer. Typically, the film is thinner at the edges than in the central region. We selected both edge and central areas and evaluated their photoelectronic properties. A direct comparison of their performance is presented in Fig. S22b. The transfer characteristic curves for the edge and central regions are displayed in Fig. S22c, d, respectively. The thinner film at the edge exhibits a larger hysteresis window compared to the thicker film in the central region. To investigate the influence of In₂Se₃ crystallinity on the heterojunction device performance, two areas with distinct crystallinity were selected for photoelectronic and ferroelectric characterization. As shown in Fig. S23a, the continuous In₂Se₃ film within the red box exhibits superior crystallinity, while the film with holes in the blue box shows inferior crystallinity. The specific photoelectronic performance data, shown in Fig. S23b, indicate that enhanced crystallinity of the In₂Se₃ film results in improved photoelectronic performance of the heterojunction device. The transfer characteristic curves for the films with different crystallinity are presented in Fig. S23c, d. The device with better crystallinity demonstrates a larger hysteresis window. Furthermore, Fig. S24 shows the specific transfer characteristic curves for the five different In₂Se₃ film regions mentioned above. Their hysteresis windows are 40, 36.6, 36.2, 33.8, and 36.8 V, respectively, yielding an average value of 36.68 V and a variance of 3.9136.

### Flame Detection System

**Fig. 3:**
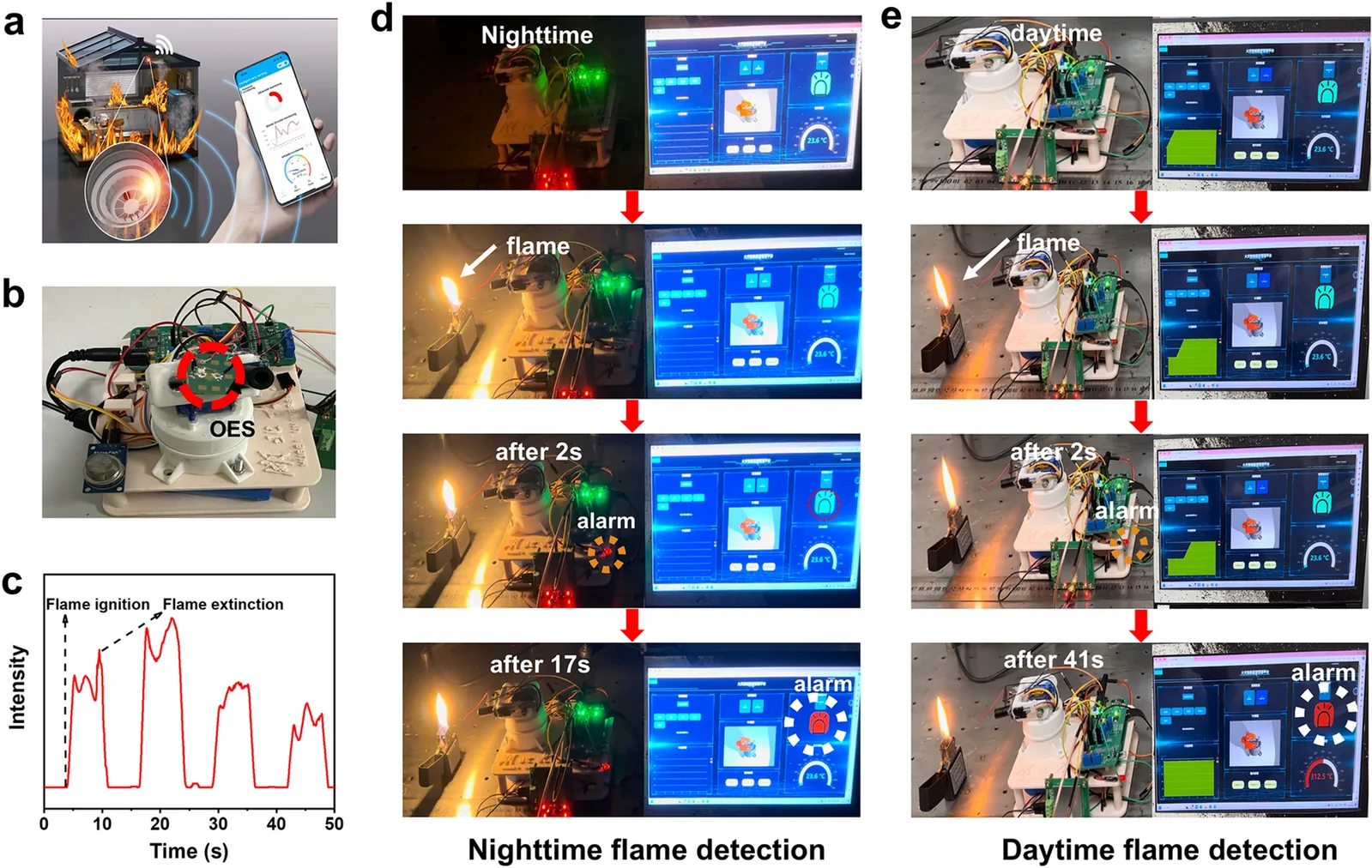
Flame detection system based on Fe-OES devices. **a** Schematic of flame detection. **b** Actual flame detector devices system. **c** Specific data on the device’s flame detection. **d** Flame alarm system and mobile alarm interface in nighttime conditions (Movie S1). **e** Flame alarm system and mobile alarm interface in daytime conditions (Movies S2 and S3)

A schematic diagram of the flame detection setup is shown in Fig. 3a. We assembled a large-area heterojunction device and connected it to a custom-built external flame alarm circuit system, as shown in Fig. 3b. The detected flame signals were amplified using multistage amplifiers in the external circuit, followed by second-order low-pass filtering and software-based mean filtering to generate the final data. The flame detection system triggers an alarm, sends the data via serial communication to the NB-IoT module, processes it, and uploads it to a cloud server for display on mobile devices. A detailed circuit schematic is provided in Fig. S25. Figure 3c presents specific data on the flame detection capabilities of the device. The fast response time and excellent detection performance of device allow it to accurately identify flame flickers during detection. The flame detection system exhibits excellent performance in both daytime and nighttime conditions. Figure S26 illustrates the flame detection results for both scenarios, showing that the alarm indicator’s rapid response time is nearly identical during detection. In the microcontroller system, a filtering algorithm has been implemented to ensure that flame detection is triggered only when signals are consistently identified. The actual response speed of the detector exceeds the detection rate configured in the microcontroller system. Additionally, traditional flame detection alarm systems can only issue alerts locally through the device itself. The conventional flame detection systems have the inherent operational limitations, even though they are sensitive to identify early fire development, this can lead to delays in emergency response in unoccupied environments or where audible alarms go unnoticed. Our flame alarm system addresses this system gap by being able to utilize mobile devices to enable immediate activation of live alarms, thus ensuring awareness regardless of personnel proximity.

Figure 3d, e shows the flame alarm system and the mobile alarm interface under daytime and nighttime conditions, respectively. At night, the flame alarm system illuminates the alarm indicator within 2–3 se of flame detection, and mobile devices issue alarms within 1 min. Similar rapid alarm responses are maintained under illuminated conditions. As shown in Fig. S27, the system and mobile devices continue to issue alarms even after the flame is extinguished. A detailed demonstration of flame detection is provided in Movies S1–S3. Movies S1 and S2 demonstrate flame detection under illuminated and dark conditions, respectively. Upon flame recognition, the alarm indicator light and buzzer on the system are activated. Movie S3 shows a physical demonstration integrated with a remote IoT host system. When a flame is detected, the local system triggers an alarm and simultaneously transmits the data to a cloud server, which updates a web-based display. Please note that a slight delay in the host interface may occur due to limitations in cloud server processing speed and real-time network latency. Furthermore, to exclude interference from common ultraviolet sources, real-world flame detection tests were conducted in an outdoor environment using tissue paper, cotton, and branches /leaves to simulate fire scenarios in office, bedroom, and forest settings, as shown in Fig. S28. Five independent combustion tests were performed for each material, and all tests successfully triggered an alarm. The average alarm times for tissue paper, cotton, and branches/leaves were 13.4, 9.6, and 3.6 s, respectively. Detailed timing data are provided in Table S3, and the video recordings are available in Movies S4 and S5. The system achieved an alarm success rate of 80% within 15 s and 100% within 25 s.

### In-sensor Motion Perception and Action Recognition

**Fig. 4:**
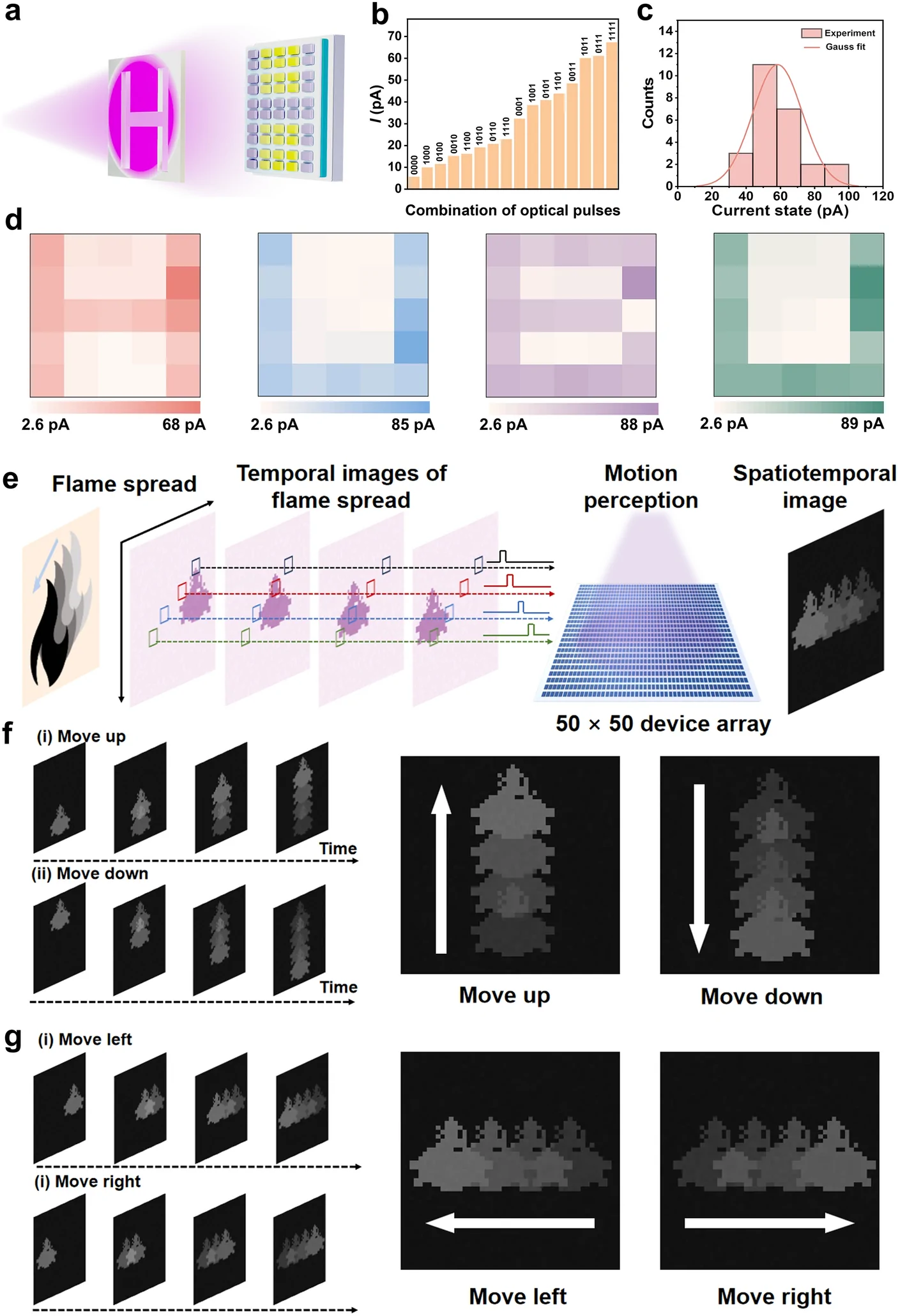
In-sensor motion perception with Ga_2_O_3_/In_2_Se_3_-based bioinspired vision sensor array. **a** Schematic of optical stimulation applied to the 5 × 5 Fe-OES array. **b** Response to the four-bit light stimulus. **c** Corresponding distribution of the photocurrent response for four consecutive pulses based on the Fe-OES array. The line represents the result of a Gaussian fit. **d** Fe-OES array under four representative light stimulation sequences ("0001," "0011," "0111," and "1111").** e** Ga_2_O_3_/In_2_Se_3_-based sensor array for mapping spatiotemporal visual information of flame spreading. **f** Output photocurrent maps for flame spreading moving (i) upward and (ii) downward. **g** Output photocurrent maps for flame spreading moving (i) leftward, and (ii) rightward

Another common limitation of traditional flame detectors is their inability to determine the extent of flame spread beyond detecting its presence, which brings significant challenges for flame motion detection with traditional flame detectors. Under continuous light pulse stimulation, the Fe-OES exhibits multi-level responses and effectively encodes temporal information at the sensing terminal, enabling potential flame motion recognition. During the experiments, 255 nm solar-blind ultraviolet light was used to simulate extremely weak flames. The process of photogenerated carriers by external optical stimulation was served to mimicking the photoconduction process of the retina. The device can assess flame propagation trends by simulating neuronal synapses. Figure 4a shows the schematic of optical stimulation applied to the 5 × 5 Fe-OES array. To evaluate the uniformity and consistency across the 25 devices, the distribution of photocurrent and dark current was measured, as shown in Fig. S29. The homogeneous distribution of both parameters among all devices indicates excellent array-level consistency. Furthermore, the optical imaging capability of the array was explored by sequentially recording the photocurrent and dark current of each device using probe scanning. The acquired data were visualized in the form of a two-dimensional current mapping (Fig. S30), which successfully resolved optical patterns corresponding to the characters “I,” “n,” “S,” “e,” “2,” and “3,” demonstrating high-quality imaging performance.

To further evaluate the feasibility of the device as a photonic synapse, we conducted a systematic study on the optoelectronic synapse properties of the Fe-OES device. As shown in Fig. S31a, when the light intensity was increased using a fixed optical pulse width of 500 ms, the device exhibited enhanced excitatory postsynaptic currents (PSC), which can be attributed to the rise in photocurrent resulting from increased carrier generation. Figure S31b displays the current response under various optical pulse durations at a fixed light intensity of 15 μW cm^−2^. Since the light intensity remains constant, the intrinsic fast response process reaches a similar level. However, longer illumination durations lead to increased trap filling, thereby enhancing only the slow decay component. Figure S31c further demonstrates the excitatory PSC response when continuous optical pulse trains are applied to the device. As the number of optical pulses increases, the cumulative effect becomes more pronounced, resulting in higher excitatory PSC responses. These optically induced dynamic responses highlight the potential of the device for in-sensor computing applications. Figure S32a shows the optoelectronic synapse response under a single 200-ms light pulse stimulation. In biological neural systems, paired-pulse facilitation (PPF) serves as a classic synaptic function, demonstrating the device’s capacity for sustained information processing. To verify the PPF effect, the device was subjected to continuous light stimulation. Figure S32b shows the light response curves for two consecutive 200 ms light pulses, with an interval of 200 ms between them. Although the two light pulses had identical power densities, the response to the second pulse was significantly larger than that to the first, indicating that the second pulse enhanced synaptic plasticity. Here, *A*_*1*_ represents the response current to the first light pulse, and *A*_*2*_ represents the response current to the second pulse. The calculated PPF index (*A*_*2*_*/A*_*1*_) was 1.46, demonstrating a strong PPF effect in the device. To further investigate this characteristic, we tested the response of the Fe-OES under four consecutive light pulse stimulations (Fig. S32c), where the response current exhibited nonlinear summation. The photocurrent of the device rises promptly upon light stimulation and decays gradually after the light is removed, rather than returning immediately to its initial state. The time interval between consecutive frames is consistent with the interval of the applied optical pulses, set at 0.25 s. The amplitude of photocurrent decay varies nonlinearly between steps. For example, after the first light exposure, the current increases from 19.3 to 27.3 pA, then decays to 24.6 pA after 0.25 s. After the second light exposure, it rises from 24.6 to 36.5 pA, then decays to 27.0 pA after 0.25 s. This nonlinear decay profile enhances the temporal feature encoding capability in our proposed pulsed encoding scheme, enabling robust and efficient representation of motion dynamics.

We treat the presence of light as a pulse "1" and the absence as "0." Under four different sequential optical pulse encodings, the conductance response of the device exhibits 16 distinct states, as illustrated in Fig. 4b. It demonstrates the distinct responses to different action frames, enabling encoding of 16 combinations for simulating and analyzing flame motion recognition in subsequent fire detection scenarios. Benefiting from the temporal encoding of the Fe-OES at the sensing terminal, the heterojunction device is capable of efficiently encoding four types of optical synaptic stimuli in real time. When the entire Fe-OES array was exposed to the same sequence of four continuous light pulses, the photocurrent variation followed a Gaussian distribution (Fig. 4c), indicating good uniformity of light stimulation. Figure 4d shows the current mapping results for the Fe-OES array under four representative light stimulation sequences ("0001," "0011," "0111," and "1111"). Additionally, the response distribution of the 25 devices to four-pulse optical stimuli is shown in Fig. S33. All devices demonstrated the capability to sequentially order the four pulses, confirming uniform synaptic functionality across the array. The Fe-OES array demonstrated excellent uniformity and stability, paving the way for subsequent flame motion recognition demonstrations.

A Ga_2_O_3_/In_2_Se_3_-based sensor array (50 × 50 pixels) was used to emulate the behavior of graded neurons. The array is capable of encoding spatiotemporal visual information and displaying trajectory contours in the visual field, enabling the perception of dynamic motion (Fig. 4e). Each device independently processes pixel-level time series data. Four temporal frames were employed to represent the spreading motion of the flame. Figure 4f, g illustrates the temporal evolution of visual stimuli corresponding to four flame spreading motions (upward, downward, leftward, rightward) applied to the 50 × 50 device array. As the spreading progresses, the photocurrent gradually decays over time after the light stimulus, with the photocurrent in the subsequent frame higher than in the previous one. The frame stacking obtained from the bioinspired sensor array consolidates spatiotemporal motion information through varying levels of photocurrent, clearly displaying the entire flame spreading trajectory from bottom to top (Fig. 4f(i)). When the spreading direction reverses, the sensor array detects the reverse trajectory contour (Fig. 4f(ii)). Additionally, Fig. 4g demonstrates the flame’s leftward and rightward spreading motion trajectories, successfully simulating the behavior of graded neurons.

**Fig. 5:**
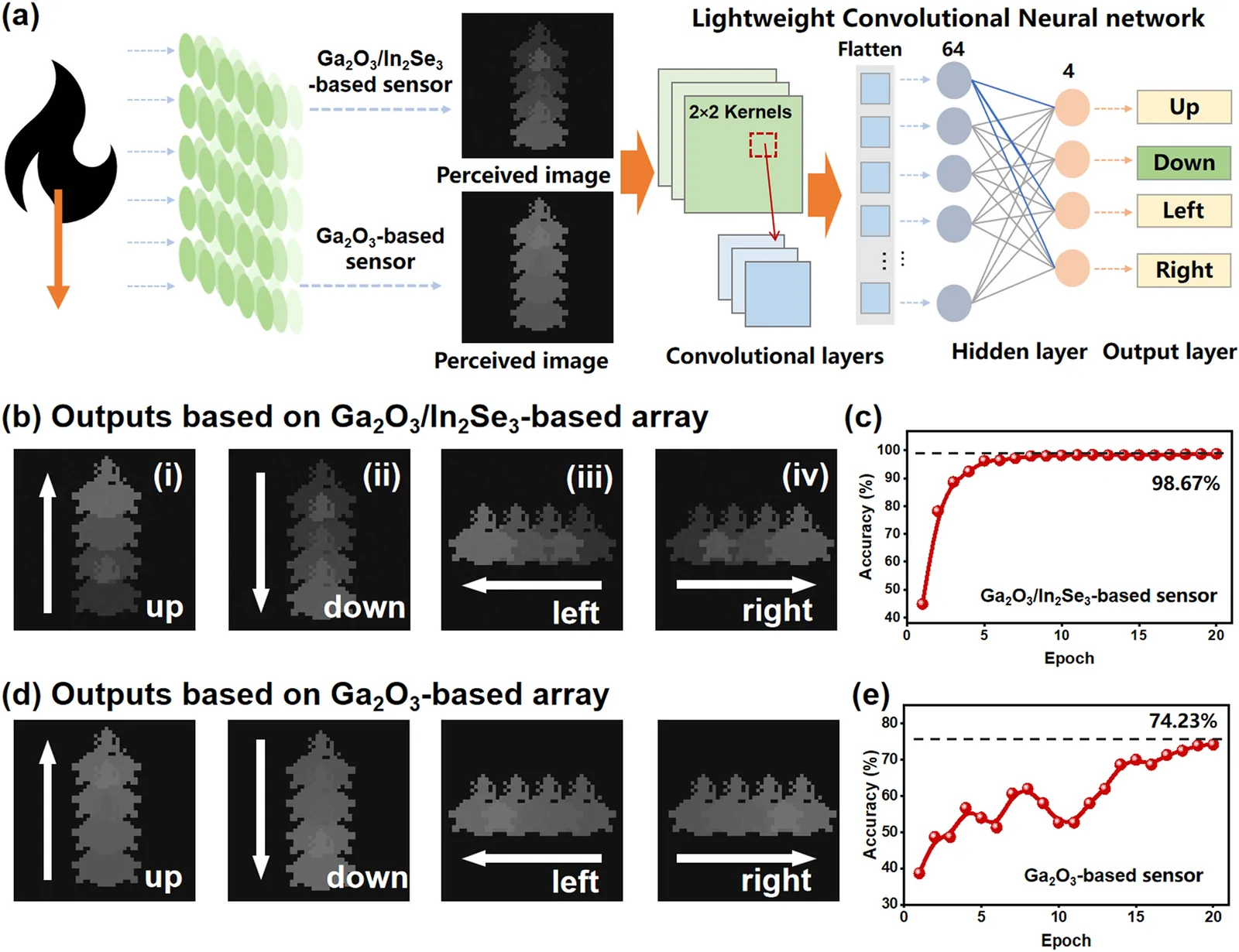
Action recognition of the Ga_2_O_3_/In_2_Se_3_-based and Ga_2_O_3_-based vision sensors. **a** Action recognition lightweight convolutional neural network with Ga_2_O_3_/In_2_Se_3_-based and Ga_2_O_3_-based sensors. **b** Flame spreading detection output from Ga_2_O_3_/In_2_Se_3_-based sensors. **c** Action recognition accuracy of Ga_2_O_3_/In_2_Se_3_-based sensors. **d** Flame spreading detection output from Ga_2_O_3_-based sensors. **e** Action recognition accuracy of Ga_2_O_3_-based sensors

By integrating temporal and spatial information, the graded neurons behavior significantly boosts the processing capability for dynamic motion recognition in machine vision applications. In this study, bioinspired in-sensor motion perception techniques based on Ga_2_O_3_/In_2_Se_3_-based and Ga_2_O_3_-based bioinspired vision sensor were utilized to classify the motion directions of flame spread (Fig. 5a). First, a custom dataset consisting of four types of flame spreading motions (upward, downward, leftward, rightward) was first developed and used to train the vision system. A lightweight convolutional neural network (CNN) was then constructed to identify the flame spreading directions, comprising a convolutional layer with eight kernels of size 2 × 2 and a fully connected layer with 64 neurons. The bioinspired vision sensor fuses spatiotemporal information from a series of frames into compressed images, which are subsequently input into a small fully connected artificial neural network for spreading direction recognition. Figure 5b illustrates the four representative flame spreading motions (upward, downward, leftward, and rightward). The Ga_2_O_3_/In_2_Se_3_-based sensor outputs frames with compressed temporal states, clearly delineating the trajectory contours to facilitate the motion recognition. Figure 5c demonstrates the recognition accuracy of the CNN of the Ga_2_O_3_/In_2_Se_3_-based sensors during training. As a result, the motion recognition accuracy achieves 98.67% after 20 epochs of training. Figure 5d presents the detection results of flame spreading using Ga_2_O_3_-based sensors. Although Ga_2_O_3_-based sensors exhibit typical photocurrent decay after continuous light exposure, the photocurrent variation between successive frames is minimal, ultimately resulting in unordered encoding. This phenomenon inherently diminishes the spatiotemporal perception capability of the pure Ga_2_O_3_-based sensors. Figure 5e shows that the recognition accuracy of the CNN based on Ga_2_O_3_-based sensors reaches 74.23% after 20 iterations, significantly lower than that of the Ga_2_O_3_/In_2_Se_3_-based sensor. To further evaluate performance, we have included a confusion matrix, as shown in Fig. S34, which demonstrates high per-class accuracy with particularly strong recognition of Ga_2_O_3_/In_2_Se_3_-based sensors.

### Neuromorphic Perception Systems

**Fig. 6:**
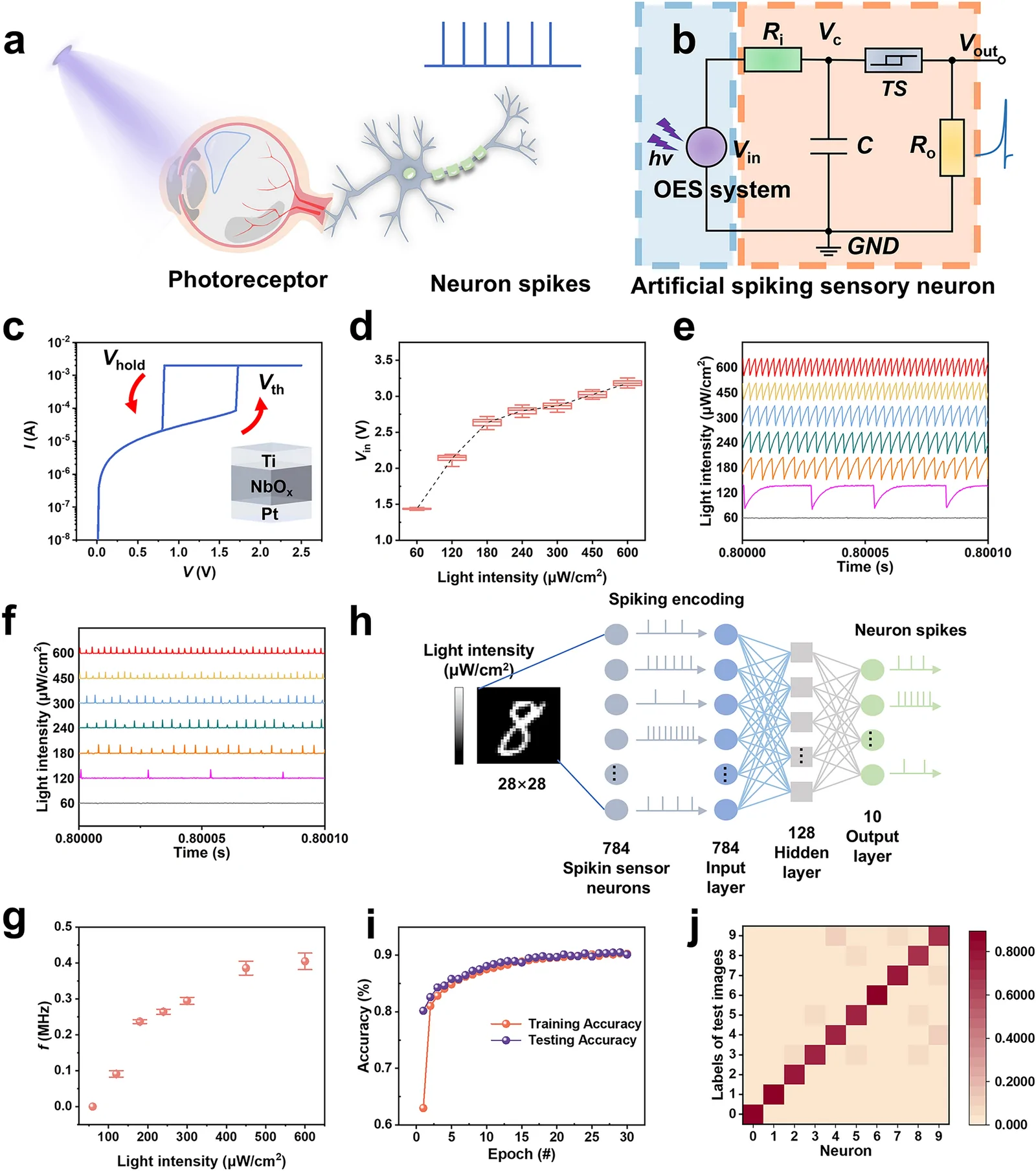
Illustration of the SNN-based system for optical perception. **a** Schematic diagram of how humans receive external light stimuli through receptors. **b** Schematic of artificial spiking vision sensory neuron. The TS device is connected in series with the output resistance, designated as *R*_o_, followed by a parallel connection with a capacitor, and finally in series with the synaptic resistance, designated as *R*_i_. **c** structural diagram and typical *I–V* characteristics of the fabricated niobium oxide threshold switching device. **d** Light-driven response currents through Fe-OES. **e** The effect of light intensity on the capacitance oscillation frequency. **f** Spike response frequency of the photonic artificial neuron under different light intensities. **g** Statistical graph of spike response frequency variation with light intensity. **h** Schematic diagram of a pulse neuromorphic sensory computing system for light intensity image recognition based on MNIST. **i** Evolution of the test accuracy with training epochs. After 30 epochs of training, the accuracy on the test set can reach 90.51%. **j** Confusion matrix of image recognition results based on the test dataset and the input light intensity images

Neuromorphic perception systems can directly interact with and efficiently process analog signals from real-world environments, offering vast potential for the development of intelligent flame detection sensors. As shown in Fig. 6a, inspired by the way humans receive external signals through receptors and encode them into spike pulses transmitted to the brain for learning and perception, we developed a photo-driven, photosensitive artificial neuron based on a Fe-OES device to effectively encode light signals from flames into spike pulses. Figure 6b illustrates the circuit diagram of the photosensitive artificial neuron, where the photo-driven response current in the Fe-OES device is converted into a voltage input for the neuron circuit via an amplifier, thereby inducing switching in the niobium oxide (NbO_x_) threshold switching device and triggering capacitor oscillations. This process results in the generation of continuous spikes at the output, mimicking the responses of biological neurons to encode and transmit sensory information. In the SNN system, the photosensitive LIF neuron is implemented in hardware, which comprises a Ga₂O₃/In₂Se₃ heterojunction device, an NbOₓ device, and interconnected resistors and capacitors, with the specific configuration shown in Fig. S35. This system converts real optical signals into neuronal spike signals. Figure 6c presents the structural diagram and typical *I–V* characteristics of the fabricated NbO_x_ threshold switching device. Due to the metal–insulator transition characteristics, the NbO_x_ device switches from a high-resistance state (*R*_*off*_) to a low-resistance state (*R*_*on*_) at Vth and reverts from a low-resistance state to a high-resistance state at *V*_*hold*_. To evaluate the sensory capability of the photosensitive artificial neuron, the output spike frequency is reliably controlled by adjusting the flame light intensity detected by the Fe-OES device. In practical experiments, weak ultraviolet light is used to simulate the early stage of flame combustion. As shown in Fig. S36a over 100 consecutive voltage sweeps, the NbOₓ threshold switching device exhibits typical threshold switching (TS) behavior: it transitions from a high-resistance state to a low-resistance state when the sweep voltage reaches the threshold voltage (~ 1.8 V), and returns to the high-resistance state as the voltage decreases to the holding voltage (~ 0.9 V). To evaluate the distribution of switching voltages, the coefficient of variation (CV) was employed as a key metric (Fig. S36b). The CV effectively reflects the degree of data dispersion, and the small CV value observed here indicates excellent uniformity in the switching voltage. Furthermore, the switching resistance distribution remains highly stable over 100 cycles (Fig. S36c). Notably, the wide voltage window (~ 0.9 V) is advantageous for applications in artificial neurons.

Figure 6d shows the variations in input voltage intensity after the conversion of the photo-driven response current by the Fe-OES device. This effect is attributed to the modulation of the Fe-OES response current by light intensity. As the incident light power increases from 60 to 600 μW cm^−2^, the input voltage of the circuit gradually increases. When the voltage *V*_*DD*_ is applied, the membrane voltage of the capacitor begins charging, as most of the voltage drop occurs across the NbO_x_ device (*R*_*off*_ > *R*_*L*_). Once the voltage exceeds *V*_*th*_, the NbO_x_ device switches to *R*_*on*_, and due to the reduced voltage drop across the NbO_x_ device (*R*_*on*_ < *R*_*L*_), the capacitor begins to discharge. Once the membrane voltage drops below *V*_*hold*_, the NbO_x_ device switches back to *R*_*off*_. This charging and discharging process repeats, inducing the neuron to continuously emit spikes, similar to biological neurons. The magnitude of the input voltage affects the capacitor’s charging and discharging time, thereby altering the operating frequency of the threshold switching device and ultimately influencing the spike frequency of the neuron. Specifically, the charging time of the neuron is determined by Eqs. (4) and (5) [53]:

4 $$\begin{array}{*{20}c} {t_{{{\mathrm{rise}}}} = - R_{r} C \times \log \left( {\frac{{V_{th} - V_{DD} \frac{{R_{r} }}{{R_{L} }}}}{{V_{hold} - V_{DD} \frac{{R_{r} }}{{R_{L} }}}}} \right)} \\ \end{array}$$

where *R*_*r*_ = *R*_*L*_||*R*_*off*_. When *R*_*out*_ is much smaller than* R*_*on*_ and can be ignored.

5 $$\begin{array}{*{20}c} {t_{{{\mathrm{fall}}}} = - R_{f} C \times \log \left( {\frac{{V_{{{\mathrm{hold}}}} - V_{DD} \frac{{R_{f} }}{{R_{L} }}}}{{V_{th} - V_{DD} \frac{{R_{f} }}{{R_{L} }}}}} \right)} \\ \end{array}$$

where *R*_*f*_ = *R*_*L*_||*R*_*off*_. If we assume that *R*_*off*_ ≫ *R*_*L*_ ≫ *R*_*on*_, then *R*_*r*_ ≈ *R*_*L*_ and *R*_*r*_ ≈ *R*_*on*_, which results in *t*_*rise*_ being proportional to *R*_*L*_ and *t*_*fall*_ being a constant significantly smaller than *t*_*rise*_. The ideal oscillation frequency *f* can be derived by utilizing Eq. (6):

6 $$f = \frac{{W_{L} }}{{C \times \log \left( {\frac{{V_{{{\mathrm{hold}}}} - V_{DD} }}{{V_{th} - V_{DD} }}} \right) }}$$

where *W*_*L*_ = 1/*R*_*L*_ represents the weight of the RRAM synapse, and *f* is directly proportional to *W*_*L*_.

Therefore, the neuron cannot generate a pulse. Different voltages are applied to the neuron, inducing capacitor oscillations at different frequencies, as shown in Fig. 6e. This results in distinguishable spike response frequencies of the photosensitive artificial neuron under different light intensities, as shown in Fig. 6f. Figure 6g shows the spike response frequency of the photosensitive artificial neuron as a function of light intensity. As the light intensity increases, the response frequency also increases. When the incident light power increases from 120 to 600 μW cm^−2^, the pulse frequency rises from 0.09 to 0.39 MHz, enabling effective perception and transmission of weak-light information. More comprehensive data can be found in Figs. S37 and S38. Additionally, long-term stability tests were conducted on the LIF neuron system (Fig. S39). Under various optical intensities ranging from 120 to 600 μW cm^−2^, the system produced up to 20,000, 100,000, 125,000, 140,000, 190,000, and 195,000 output spikes, respectively. The variation in spike counts across light intensities is due to differences in the pulse frequency generated by the integration-and-fire (LIF) neuron per unit time under different stimulation conditions. Even after such high numbers of operational cycles, no significant degradation in device-to-device performance was observed, demonstrating excellent consistency and stability across devices.

This weak-light perception and encoding capability can be further applied to neuromorphic computing systems. Figure 6h illustrates the schematic diagram of a pulse neuromorphic sensory computing system for light intensity image recognition based on Modified National Institute of Standards and Technology (MNIST) database. In this system, each pixel value in the handwritten digit image is considered as relative light intensity. A total of 784 photosensitive artificial neurons are used to perceive light intensity and encode it into pulses of different frequencies. The encoded spike sequence is processed by a spike neural network consisting of 784 input neurons, 196 hidden neurons, and 10 output neurons. As shown in Fig. 6i, after 30 training iterations, the image recognition accuracy for test data reaches 90.51%. Figure 6j presents the confusion matrix of image recognition results based on the test dataset and the input light intensity images. Almost all digit images can be correctly classified, further confirming the ability of this photosensitive artificial neuron system to process the extremely weak light generated in the early stages of flame combustion through a neuromorphic perception system, demonstrating its immense potential for applications in tasks such as fire warning systems and missile plume tracking.

## Conclusions

We developed a Fe-OES system based on Ga_2_O_3_/In_2_Se_3_, where a single device can exhibit multi-operational modes of "all-or-none" pulse characteristics and time-sum characteristics under continuous pulse stimulation by gate regulation. A 5 × 5 Fe-OES array fabricated using this device demonstrates high uniformity and stability in response to light stimulation. The Fe-OES system effectively achieves three representative tasks, showcasing its multi-platform, multi-task application advantages.

Through low-level sensing processing, it efficiently handles flame information such as noise reduction and feature enhancement, enabling efficient flame detection and alarming over the entire time frame. For advanced processing, the Fe-OES system effectively encodes temporal information via in-sensor computing, achieving accurate simulation of flame motion recognition. Using an ultra-lightweight neural network with just 64 neurons, it achieves an impressive recognition accuracy of 96.47%. Additionally, we demonstrated a photosensitive artificial neural system based on Fe-OES that can effectively process extremely weak light from the early stages of flame combustion via neuromorphic perception, achieving a recognition accuracy of 90.51%. The multi-platform, multi-task application advantages of the Fe-OES system can significantly advance the development of complex tasks such as flame detection, fire warning, and missile exhaust plume tracking.

## Supplementary Information

Below is the link to the electronic supplementary material.

Supplementary file1 (MP4 1970 KB)

Supplementary file2 (MP4 2497 KB)

Supplementary file3 (MP4 3056 KB)

Supplementary file4 (MP4 1602 KB)

Supplementary file5 (MP4 1316 KB)

Supplementary file6 (DOCX 11295 KB)
