# Supplementary material for: Ferroelectric Optoelectronic Sensor for Intelligent Flame Detection and In-Sensor Motion Perception
Source: Nanomicro Lett. 2026 Jan 13;18:123. doi: 10.1007/s40820-025-01968-x (PMC12796090; doi:10.1007/s40820-025-01968-x)
Supplement: Supplementary file 6 — Supplementary file6 (DOCX 11295 KB) [file 40820_2025_1968_MOESM6_ESM.docx]

Supporting Information for

**Ferroelectric Optoelectronic Sensor for Intelligent Flame Detection and In-Sensor Motion Perception**

Jiayun Wei^1,#^, Guokun Ma^1,#^, Runzhi Liang^1,#^, Wenxiao Wang^2,#^, Jiewei Chen^2^, Shuang Guan^1^, Jiaxing Jiang^1^, Ximo Zhu^1^, Qian Cheng^1^, Yang Shen^1^, Qinghai Xia^1^, Shiwen Wu^1^, Houzhao Wan^1^, Longhui Zeng^3,^*, Mengjiao Li^4^, Yi Wang^5^, Liangping Shen^1,^*, Wei Han^1,^*, Hao Wang^1,^*

^1^Institute of Microelectronics and Integrated Circuits, School of Integrated Circuits, Hubei University, Wuhan 430062, P. R. China

^2^School of Physics, South China Normal University, Guangzhou 510006, P. R. China

^3^School of Physics and Microelectronics, Key Laboratory of Material Physics Ministry of Education, Zhengzhou University, Zhengzhou 450052, P. R. China

^4^School of Microelectronics, Shanghai University, Jiading, Shanghai 201800, P. R. China

^5^Center for Microscopy and Analysis, Nanjing University of Aeronautics and Astronautics, Nanjing 210016, P. R. China

#Jiayun Wei, Guokun Ma, Runzhi Liang, and Wenxiao Wang contribute equally to this work.

*Corresponding authors. E-mail: [wangh@hubu.edu.cn](mailto:wangh@hubu.edu.cn) or [nanoguy@126.com](mailto:nanoguy@126.com) (Hao Wang); [weihan@hubu.edu.cn](mailto:weihan@hubu.edu.cn) (Wei Han); [lpshen@hubu.edu.cn](mailto:lpshen@hubu.edu.cn) (Liangping Shen); [lhzeng.hfut@gmail.com](mailto:lhzeng.hfut@gmail.com) (Longhui Zeng)

**S1 Growth of Ga_2_O_3_ films and annealing**

The synthesis of Ga_2_O_3_ thin films was achieved through atomic layer deposition (ALD). The specific process involved the introduction of a clean and dry Si substrate, coated with a 300 nm SiO_2_ insulating layer, into the chamber of the ALD equipment. The substrate was subjected to oxygen plasma for a period to precondition the surface and remove contaminants from the substrate surface. Trimethylgallium was deposited for 0.1 s, followed by 20 s of argon purging, and then 10 s of oxygen exposure. The typical growth temperature is about 300℃.

**S2 Growth of In_2_Se_3_ films**

Approximately 100 mg of In_2_Se_3_ powder was placed in a quartz boat located at zone 2 of the heating area, evenly spread out. Commercial mica (KMg_3_AlSi_3_O_10_F_2_) sheets were used as substrates, placed directly above the In_2_Se_3_ powder, and the vertical distance between the source material and the substrate was controlled within a narrow range (approximately 3 mm). Prior to the reaction, the system was evacuated under preliminary vacuum, followed by maintaining a vacuum level of approximately 0.5 Pa. The target temperature of the heating zone was typically set to 800 °C. After reaching the target temperature, the holding time was typically 30 minutes. The heating rate was typically 33 °C/min. After the reaction, the quartz boat was slowly pulled out of the heating zone using a magnet, and the product cooled down to room temperature in the furnace, resulting in a 2D In_2_Se_3_ film on the mica substrate.

**S3 Transfer method of In_2_Se_3_ films**

First, the surface of mica sheets grown with two-dimensional phase In_2_Se_3_ films was coated with a layer of PMMA solution using a spin coater at 2000 rpm. Subsequently, the PMMA-coated samples were baked on a hot plate at 80 °C for 5 minutes to evaporate the organic solvent in the PMMA, thereby solidifying it into a film. The samples were then surrounded with tape, immersed in deionized water, and using tweezers, the PMMA film containing the sample was peeled off from the mica due to the surface tension of the water. Next, the PMMA film with the sample was transferred onto a Ga_2_O_3_ film and baked at 45℃ on a hot plate for 10 minutes to completely evaporate the water. After dissolving the PMMA with acetone solution, the In_2_Se_3_ film was transferred onto the Ga_2_O_3_ film.

**S4 Materials characterizations**

The In_2_Se_3_/Ga_2_O_3_ heterojunction was characterized by OM (Ningbo Sunny, CX40M), KPFM and PFM (Oxford Instruments Asylum Research and Cypher S), XPS (Thermo Fisher Scientific K-Alpha) all samples were analyzed using an Al Ka X-ray source (spot size of 400 um) at a constant dwelling time for 100 ms wide scan (single scan, a step size of 1eV) and 300 ms narrow scan (5 scans, a step size of 0.05 eV). The survey spectra and high-resolution single core-level spectra were measured at the pass energies of 150 and 30 eV, respectively. To neutralize the charge on the sample during the experiments, an electron-ion charge compensation system was used. The studies were carried out under ultrahigh vacuum 10^-9^ mba at room temperature; in the case of using a sample charge compensation system, the partial pressure of argon in the analytical chamber was 5 × 10^-7^ mbar. The experimental data were processed using Thermo Fisher Scientific K-Alpha, the spectrometer sofware (Avantage Thermo Fisher Scientific). The cross-section sample was made by focused-ion beam (FIB, Thermo Fisher Helios5 UX), and the TEM/EDS data was collected by a spherical aberration-corrected TEM (JEOL JEM-ARM200F).

**S5 Device fabrication and characterization**

Au electrodes were deposited by a thermal evaporation coater by using TEM grids (Zhongjingkeyi) as hard masks. Following the removal of the grids, the Au electrodes were fabricated. All current-voltage (*I-V*) and current-time (*I-t*) curves were measured in a vacuum environment in the probe-station (MyProber) using the semiconductor analyzer (Keithley 4200-SCS). The noise current is measured by PDA PXle-FS380 and Keithley 4200A-SCS. The light source used for the measurement was a LED 255 nm whose optical power was measured by a standard silicon photodiode and adjusted by a neutral density optical filter.

**S6 Flame detection alarm system**

The system comprises an In_2_Se_3_/Ga_2_O_3_ heterojunction flame detector, a smoke detector, a detector scanning platform, a detector *I-V* amplifier circuit, an ESP-Wroom-32 master control circuit, and an NB-IoT communication circuit. The specific operation principle is as follows: When a flame is detected, the flame detector senses it, causing a change in the detector's current. This changed current then passes through the *I-V* amplifier circuit. The ESP-Wroom-32 master control unit reads the amplified voltage signal and subsequently transmits the information via WiFi or the NB-IoT network.

**S7 CNN graded neuron computing**

In this study, the lightweight CNN was trained on a custom dataset consisting of four classes of flame spreading motions (upward, downward, leftward, and rightward), containing a total of 1000 images, with each category comprising approximately 250 images. The model architecture consisted of a single convolutional layer with 8 filters of size 2×2 using ReLU activation, followed by max pooling with a 2×2 window, a fully connected layer with 64 units, and a 4-unit softmax output layer. The model was compiled with the Adam optimizer (default learning rate of 0.001) and sparse categorical cross-entropy loss, and trained over 20 epochs with a batch size of 32. The dataset was split using a 70%/30% train-test ratio. Additionally, we conducted a energy consumption analysis focused on **software-level** computational efficiency. Our results show a total training energy consumption of 154.67 Joules for the software training process across 20 epochs. More significantly for practical deployment scenarios, the inference phase demonstrates remarkable efficiency with 5.13 Joules consumed per inference at a processing time of 0.103 seconds per images. This software-level energy analysis provides valuable insights into the computational efficiency of our algorithm when implemented on standard processing hardware.

The core innovation of our system lies in a system that efficiently couples a graded neuromorphic sensing device with backend neural network processing. Rather than focusing on algorithmic advances in the network structures themselves, our contribution is centered on how the device’s native analog encoding capability simplifies and enhances downstream digital processing. The device continuously captures visual inputs and converts them into spatiotemporally correlated conductance variations, effectively compressing raw temporal sequences into low-dimensional feature representations.

In the context of the CNN-based processing pathway (Figure 5), the device itself does not perform computation within the network, nor does it replace any neural layers or act as a dynamic synapse. The entire CNN, including its convolutional layers, activation functions, and classification layers, is implemented in software. The principal role of the device here is to serve as a sensing-computing integrated sensor that encodes temporal dynamics and spatial contrasts into analog conductance profiles. These profiles are then mapped to grayscale image frames that already incorporate meaningful motion and structural information. As a result, the CNN receives highly preprocessed inputs rather than raw pixel streams, drastically reducing the network’s architectural complexity and computational burden. In this way, the device serves as an encoding front-end rather than a computational element within the CNN.

We further emphasize that the device is solely involved in the inference stage as part of the sensing and encoding front-end. It plays no role in the training process, which means all model parameters are updated via backpropagation executed on a digital computer. To ensure reproducibility and clarify the physical–computational interface, we define the mapping from device conductance to CNN input as follows. The conductance values *G*, as measured from the device under optical stimuli, are normalized using the following equation.

*G*_Norm_=(*G*−*G*_min_)/(*G*_max_−*G*_min_)

where *G*_min_ and *G*_max_ denote the minimum and maximum conductance values observed across the entire dataset under controlled experimental conditions. This normalized value (*G*_Norm_) is subsequently scaled by 255 to generate grayscale pixel intensities.

*I*_pixel_ = |255⋅*G*_Norm_|

where *I*_pixel_ represents the grayscale pixel intensity value obtained after mapping the normalized conductance value to the standard 0-255 image range. The resulting image is then fed directly into the software-based CNN. This approach provides a transparent and reproducible bridge between the analog device response and digital network processing.

**S8 Fabrication and testing of artificial neuron hardware**

The high purity Nb_2_O_5_ target (99.999%) and TiO_2_ target (99.999%) were co-sputtered to deposit NbO_x_:Ti thin films as threshold switch (TS) layer on Si/SiO_2_/Ti/Pt substrate by RF sputtering deposition (ULVAC ACS-4000-C4). The used power for Nb_2_O_5_ and TiO_2_ is 55 W and 5 W, respectively. The co-sputtered time is 2400 s. Then, the metal Ti (99.99%) top electrodes were patterned by mask alignment process and were deposited by DC sputtering. The NbO_x_ TS device is connected in series with the output resistor (R_o_=50 Ω) and the capacitor (C=1 nF) in parallel, and the integrated input resistor (R_i_=5 kΩ) is then connected in series to construct LIF (leaky integration-and-fire) artificial neuron. Photoelectric artificial neurons were constructed by connecting Fe-OES devices with LIF artificial neurons. The electrical characteristics of the device were measured by Keysight B1500A semiconductor parameter analyzer. During electrical testing, a voltage is applied to the Ti top electrode (TE), and the Pt bottom electrode (BE) is grounded. In neuron test, the Siglent SDS2504X HD digital oscilloscope was used to observe the voltage of each circuit module during neuron behavior test in real time. The signals of input pulse V_in_, capacitor voltage V_c_ at both ends and output pulse V_out_ are detected by three channels respectively. The output trigger mode is adopted to monitor the status of each unit, access and read the required data.

**S9 SNN-based system for optical perception**

The threshold switching memristor is connected in series with the output resistor, denoted as *R_o_*, then in parallel with a capacitor, and finally in series with the synaptic resistor, denoted as *R_i_*. The input signal is converted from external solar-blind ultraviolet light into an electrical signal by the Fe-OES, which is then amplified and converted into a voltage signal by the detection system. An oscilloscope is used to measure the input, capacitor, and output waveforms. Upon applying the input voltage, the capacitor begins to charge. Once the capacitor voltage exceeds the threshold voltage, the TS device transitions from a high-resistance state to a low-resistance state. Simultaneously, the neuron generates a pulse through the output resistor. During the capacitor's discharge through the TS device and *R_o_*, any input voltage charging the capacitor is released due to the discharge. When the capacitor voltage drops below the holding voltage, the TS device reverts to its high-resistance state. At this point, the capacitor re-enters the charging state, preparing for the next discharge. The capacitor and output pulses vary according to the intensity of the input ultraviolet light.

**S10** **Simulation of SNN**

First, MNIST data is loaded through Torchvision, and then the image is preprocessed. We then build a custom frequency encoder that converts the input image pixel values into a pulse column that matches the pulse emitting frequency. The encoder calculates the transmission frequency through the exponential attenuation formula, and generates a pulse column within the specified time step (T_steps), which is provided as an input signal to the SNN. Then a SNN model consisting of two fully connected layers (Linear) and two layers of LIF neurons is constructed using the modules provided by SpikingJelly. The mean square error (MSE) is then used as a loss function to quantify the difference between the firing frequency of the output neuron and the true label (One-hot encoding). Adam algorithm is selected by the optimizer, which combines adaptive learning rate and momentum mechanism to improve the stability and convergence speed of the training process. In the training process, training was conducted on a custom dataset comprising 60,000 training images and 10,000 test images. The image data were converted into PyTorch tensors using transforms.ToTensor and normalized to the range [0, 1]. The DataLoader was utilized to load the data in batches, with a batch size of 256. The training set was shuffled to improve generalization, while the test set remained unshuffled to ensure consistent evaluation. The model was trained using the Adam optimizer, which incorporates adaptive learning rate and momentum mechanisms to enhance training stability and convergence speed. The learning rate was set to 0.001, and training proceeded for 30 epochs with a batch size of 256.

**S11 Supplementary Figure and Tables**


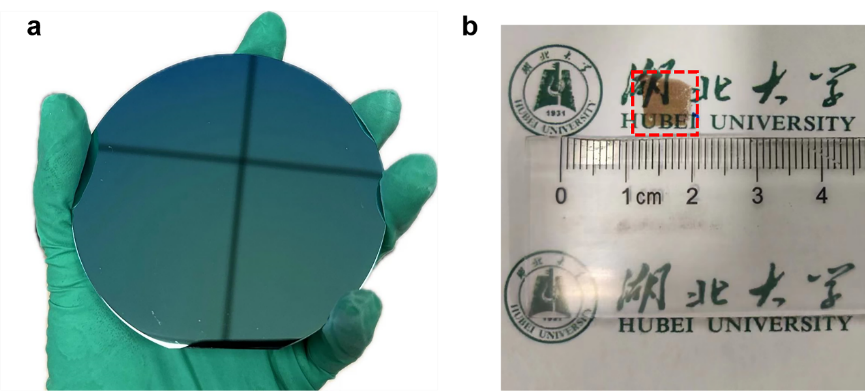


**Fig. S1 a,** Photograph of Ga_2_O_3_ thin film prepared by ALD method on four-inch Si substrate. **b,** Photograph of centimetre-scale In_2_Se_3_ film grown on mica substrate by CVD method


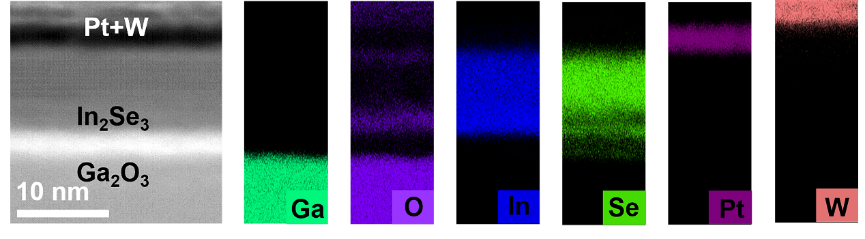


**Fig. S2** The cross-sectional TEM and EDS mapping images at the Ga_2_O_3_/In_2_Se_3_ interface


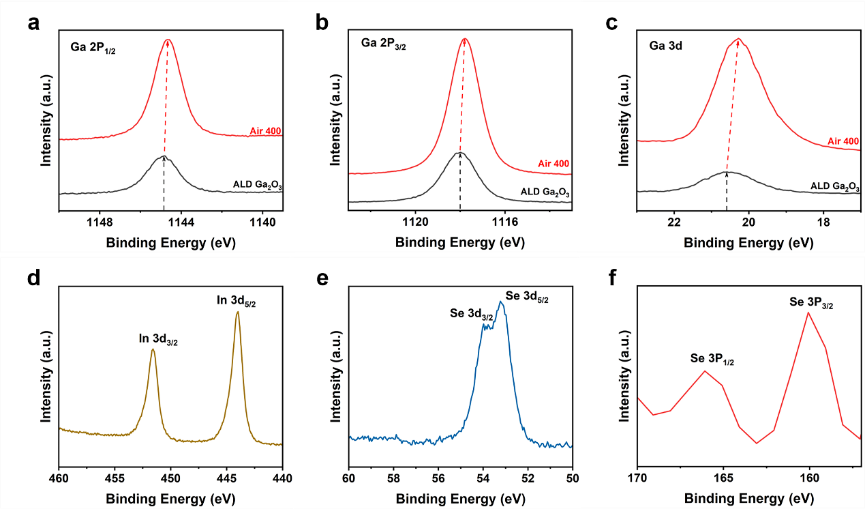


**Fig. S3 Images of changes in the binding energies of a,** Ga2p_3/2_, **b,** Ga2p_1/2_, **c,** and Ga3d for pre-annealed and annealed Ga_2_O_3_ films. Images of **d,** In3d, **e,** Se3d, and **f,** Se3p peaks of In_2_Se_3_ films


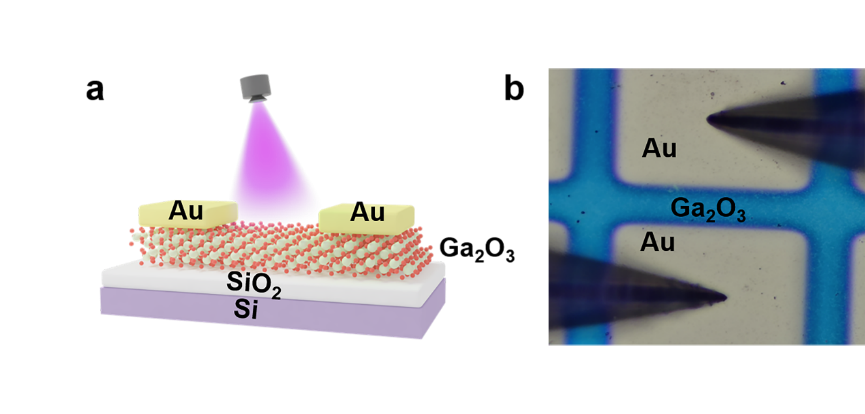


**Fig. S4 a,** structure of Ga_2_O_3_ device. **b,** Optical image of Ga_2_O_3_ device


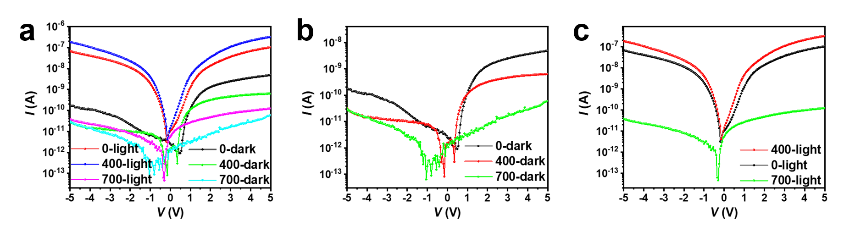


**Fig. S5 I-V curves of Ga_2_O_3_ film devices were prepared without annealing, annealed at 400 ℃ in air, and annealed at 700 ℃ in air. a,** Output characteristics of the Ga_2_O_3_ film devices in the dark and UV illumination. **b,** Dark current. **c,** Photocurrent


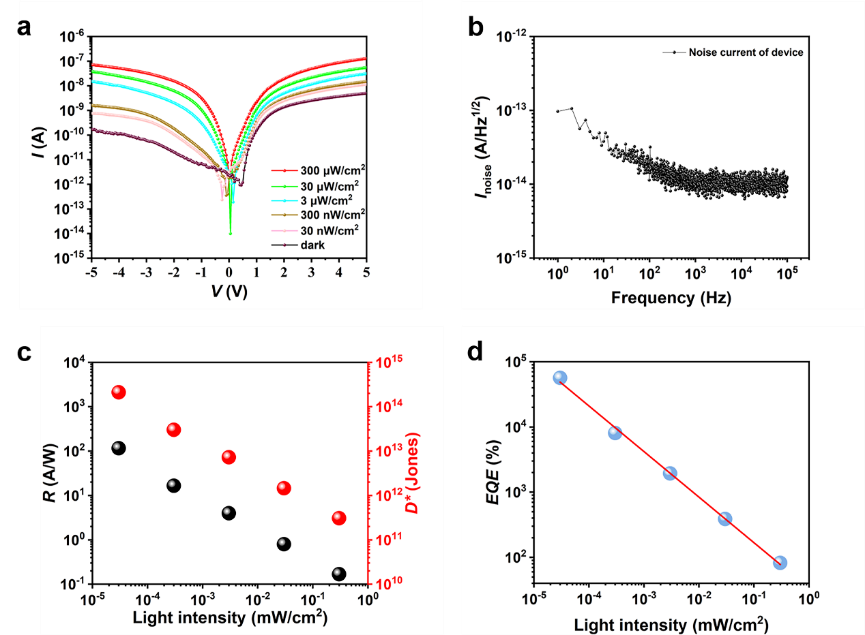


**Fig. S6 Photoelectric performance of unannealed Ga_2_O_3_ film devices. a,** Photoresponse *I–V* characteristic curves of the device. **b,** Noise current of the device. **c**, *R* and *D** of the device on the light intensity. **d,** *EQE*

The definition formula is as follows:

$$\begin{aligned} \text{EQE = }\frac{\text{R h c}}{\text{q λ}}\text{\#} \end{aligned}$$

In this formula, *h* refers to Planck's constant, *c* is the speed of light, *q* is the elementary charge, and *λ* is the wavelength of the irradiated light. A higher *EQE* value in a photodetector signifies a greater efficiency in converting incoming photons into photo-charge carriers. The external quantum efficiency (*EQE*) of the non-annealed device, as illustrated in **Fig. 6d**, is 56498%.


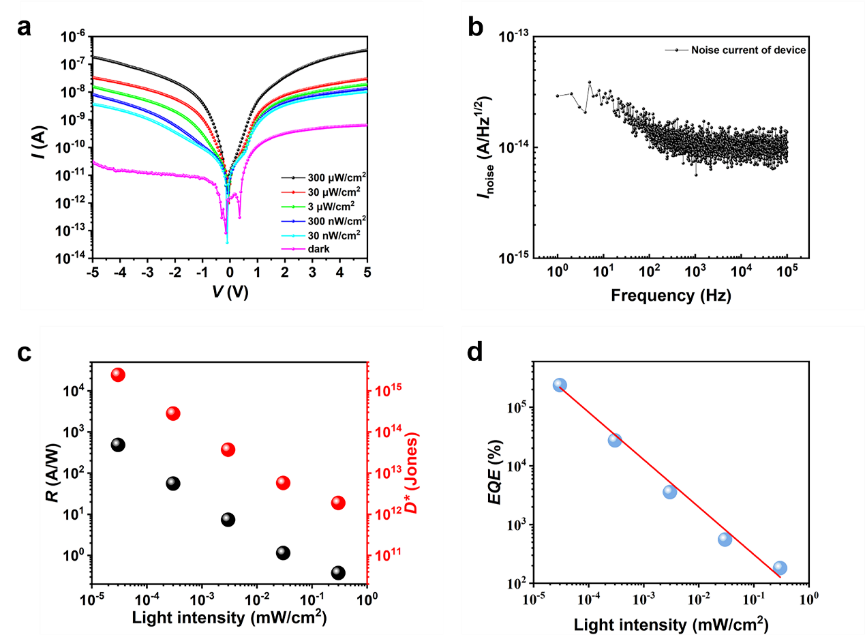


**Fig. S7 Photoelectric performance of Ga_2_O_3_ film device annealed at 400 ℃. a,** Photoresponse *I–V* characteristic curves of the device. **b,** Noise current of the device. c, *R* and *D** of the device on the light intensity. **d,** *EQE*


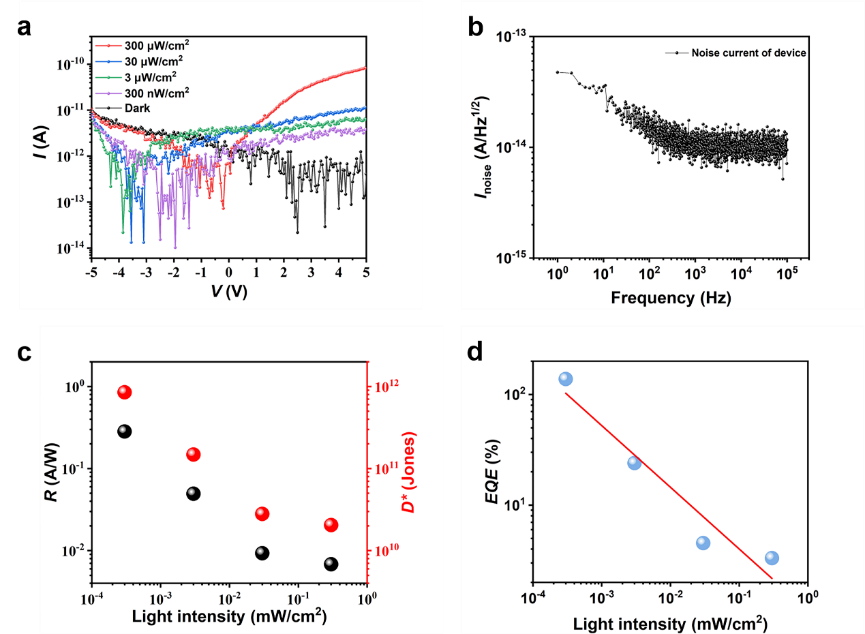


**Fig. S8 Photoelectric performance of Ga_2_O_3_ film device annealed at 700 ℃. a,** Photoresponse *I–V* characteristic curves of the device. **b,** Noise current of the device. c, *R* and *D** of the device on the light intensity. **d,** *EQE*


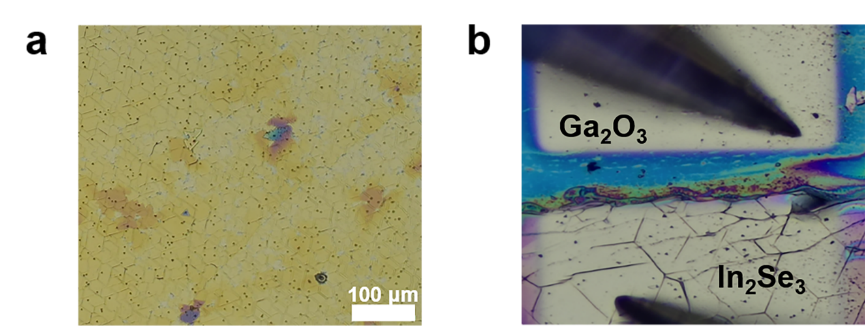


**Fig. S9 a,** OM images of In_2_Se_3_ film before transfer. **b,** OM images of Ga_2_O_3_/In_2_Se_3_ heterojunction device


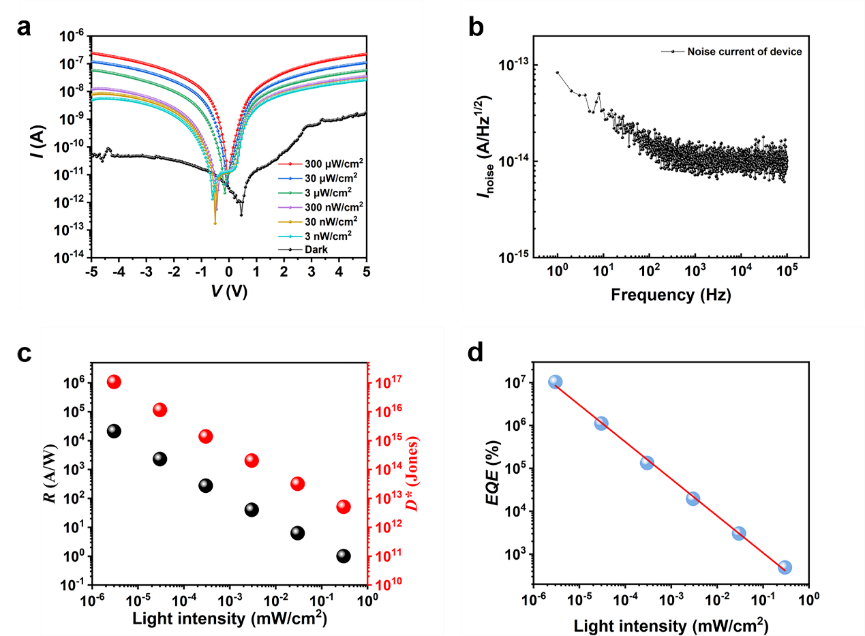


**Fig. S10 Photoelectric performance of annealed at 400 ℃ Ga_2_O_3_/In_2_Se_3_ heterojunction device. a,** Photoresponse *I–V* characteristic curves of the device. **b,** Noise current of the device. c, *R* and *D** of the device on the light intensity. **d,** *EQE*


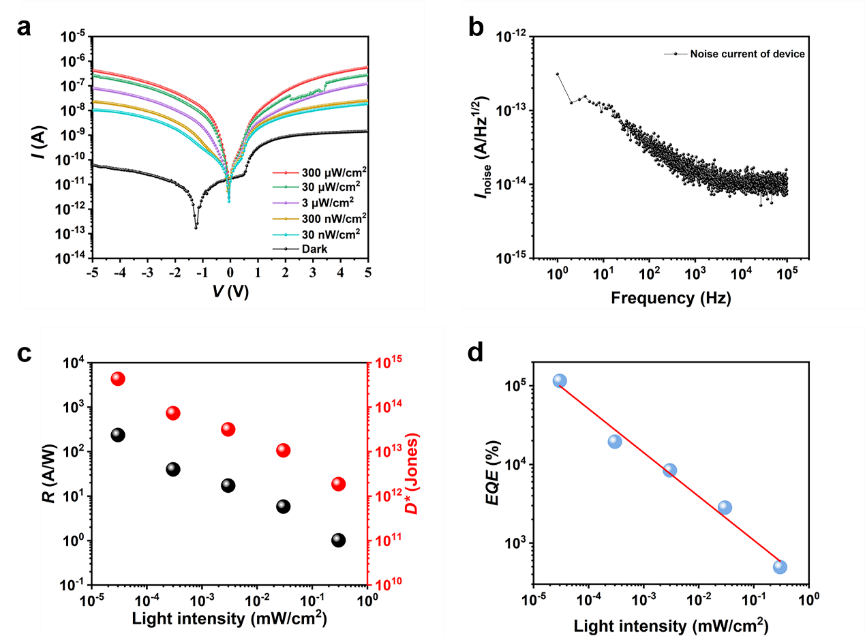


**Fig. S11 Photoelectric performance of unannealed Ga_2_O_3_/In_2_Se_3_ heterojunction device. a,** Photoresponse *I–V* characteristic curves of the device. **b,** Noise current of the device. c, *R* and *D** of the device on the light intensity. **d,** *EQE*


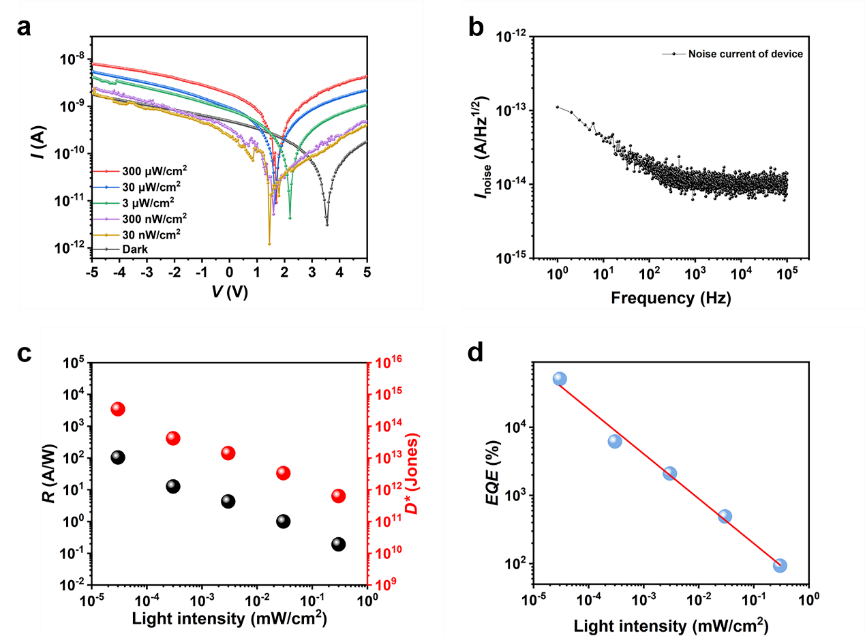


**Fig. S12 Photoelectric performance of Ga_2_O_3_ annealed at 700 ℃/In_2_Se_3_heterojunction device. a,** Photoresponse *I–V* characteristic curves of the device. **b,** Noise current of the device. c, *R* and *D** of the device on the light intensity. **d,** *EQE*


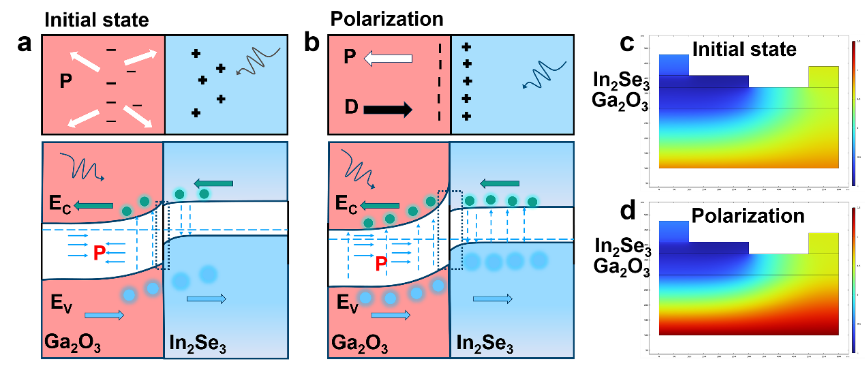


**Fig. S13** Band alignment and the photogenerated carrier transport process under **a,** in initial state condition, and **b,** in polarization condition. **c,** Electric field distribution in the initial state simulated using COMSOL Multiphysics. **d,** Electric field distribution under polarized conditions simulated using COMSOL Multiphysics


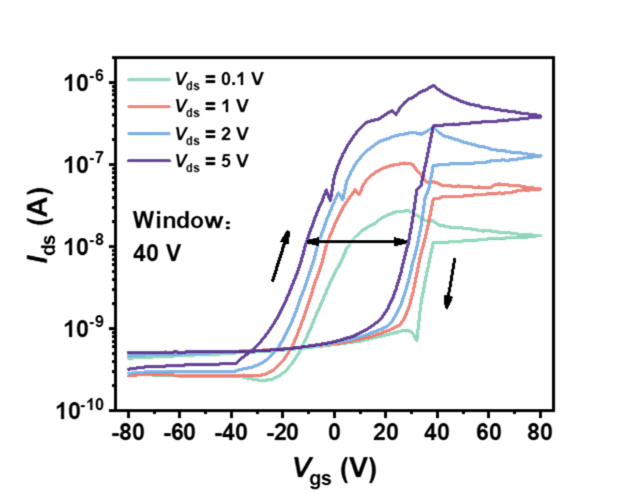


**Fig. S14** Transfer characteristics of the Ga₂O₃/In₂Se₃ heterojunction under various drain-source voltages


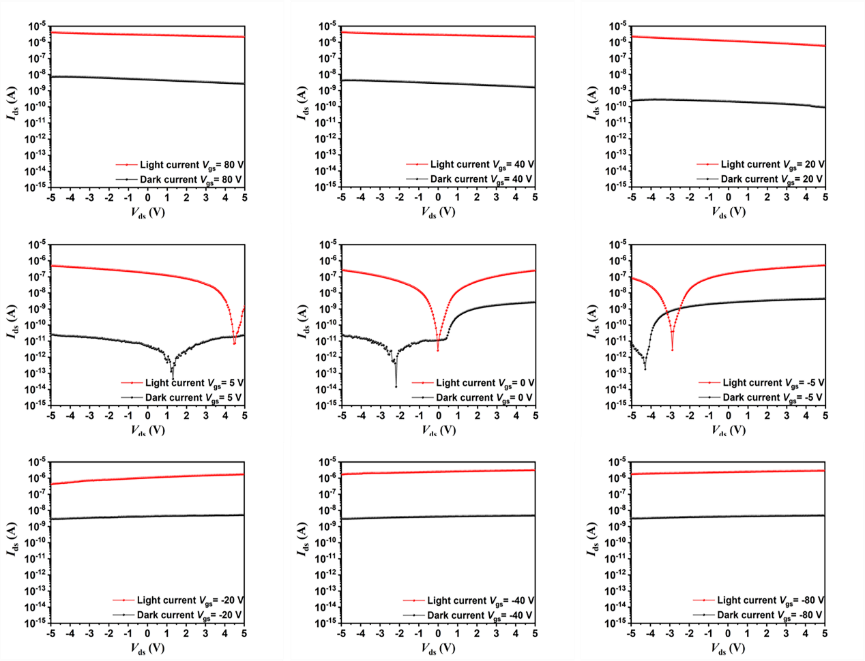


**Fig. S15** *I_ds_ – V_ds_* curves testing under different gate voltages before and after exposure to 255 nm UV light


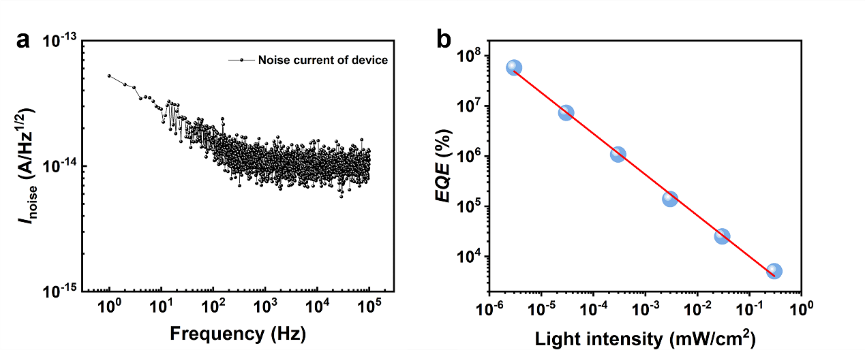


**Fig. S16** Noise current of the Ga_2_O_3_/In_2_Se_3_ heterojunction device at 5 V gate voltage


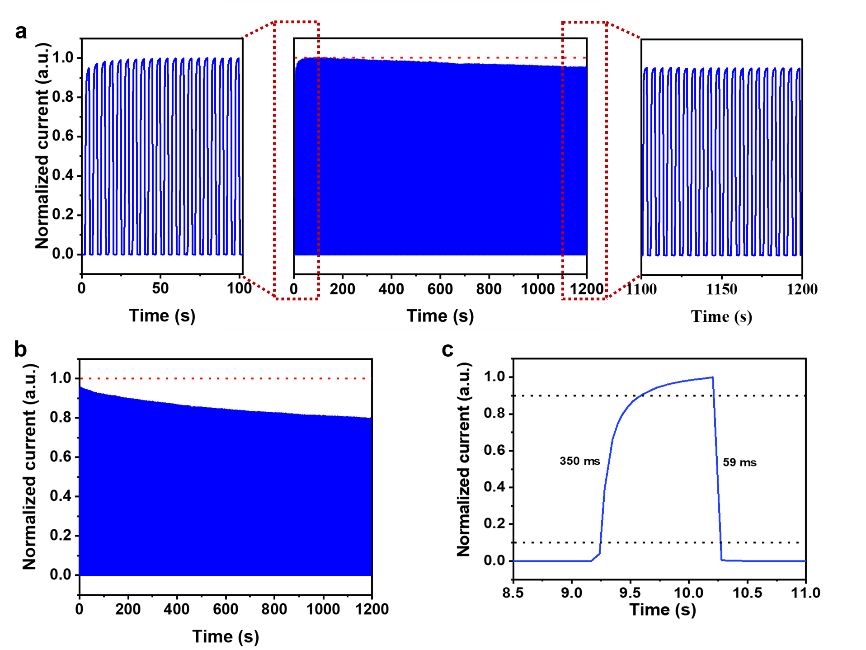


**Fig. S17 a,** Stability and reliability characteristics of the Fe-OES after 1200 s. **b,** Stability and reliability characteristics of the Fe-OES after 12h. **c,** Response time


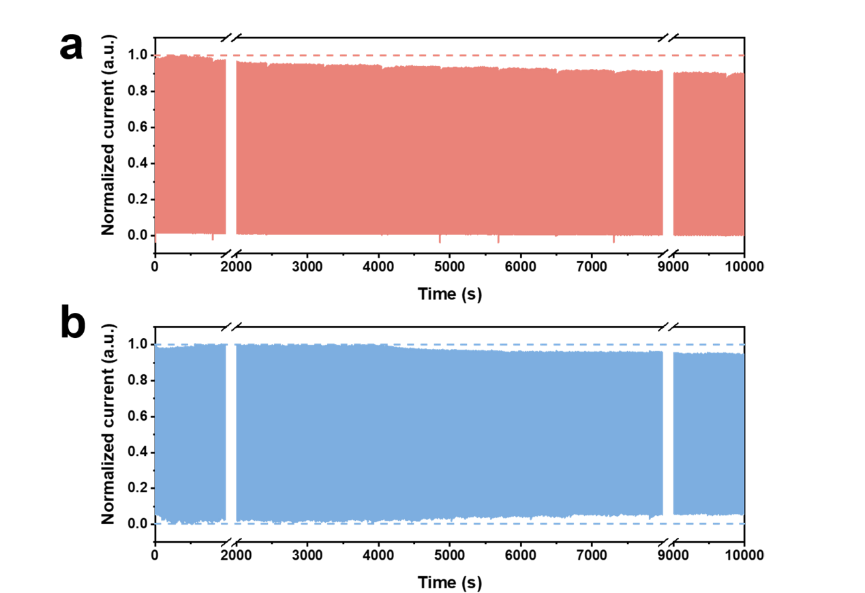


**Fig. S18 a,** Stability and reliability characteristics of the Fe-OES after 10,000 seconds under a gate voltage of 20 V. **b,** Stability and reliability of the Fe-OES after 10,000 seconds of cyclic gate voltage sweeping between -20 V and 20 V


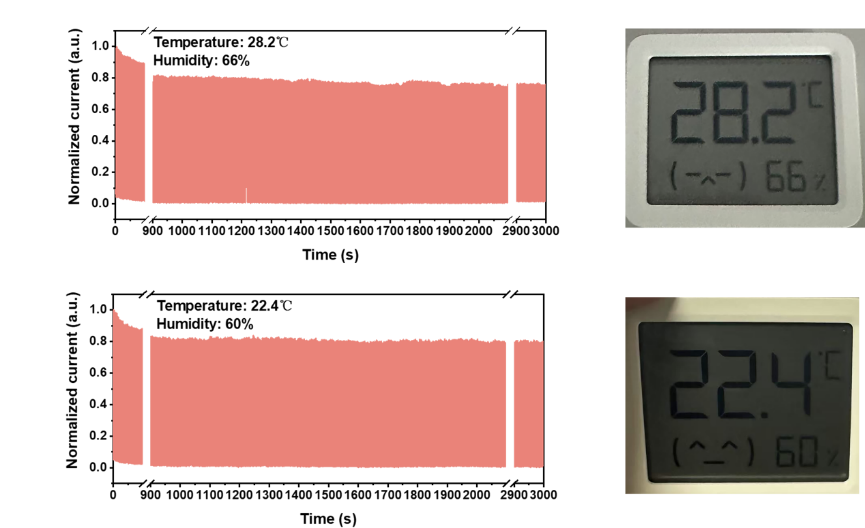


**Fig. S19** Stability testing of the device under high humidity and varying temperature conditions


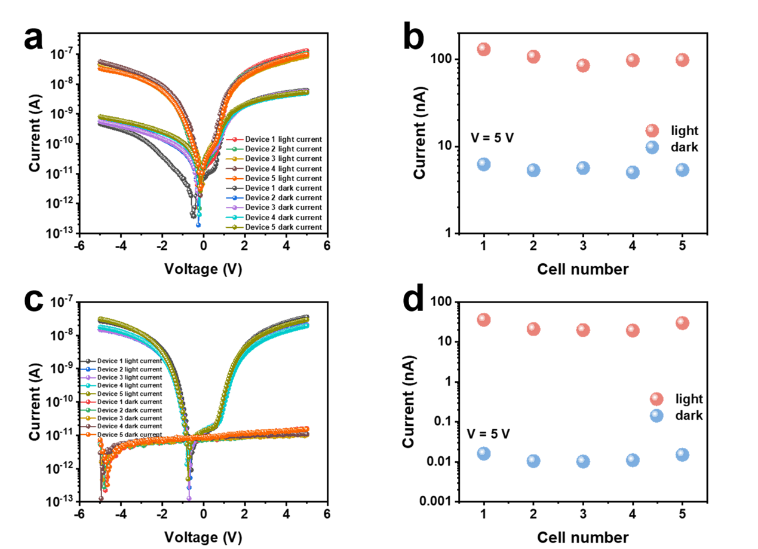


**Fig. S20 a,** Photocurrent and dark current at five distinct regions on the Ga₂O₃ film. **b,** The specific values of the photocurrent and dark current at 5 V on the Ga₂O₃ film. **c,** Photocurrent and dark current at five distinct regions on the Ga₂O₃/In₂Se₃ film. **d,** The specific values of the photocurrent and dark current at 5 V on the Ga₂O₃/In₂Se₃ film


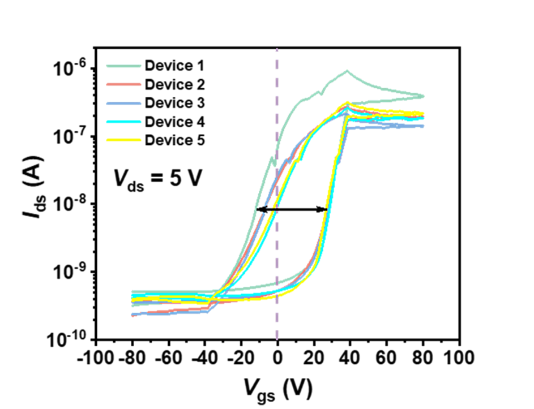


**Fig. S21** Transfer characteristic curves at five distinct regions on the Ga₂O₃/In₂Se₃ film


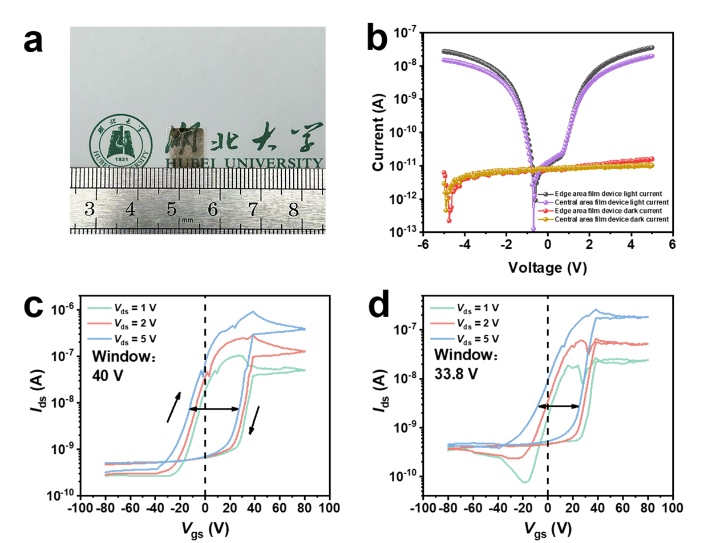


**Fig. S22 a,** Photograph of the as-grown In₂Se₃ thin films. **b,** Photocurrent and dark current at the edge and central regions of the Ga₂O₃/In₂Se₃ film. **c,** Transfer characteristic curves in the edge regions of the Ga₂O₃/In₂Se₃ film. **d,** Transfer characteristic curves in the central regions of the Ga₂O₃/In₂Se₃ film


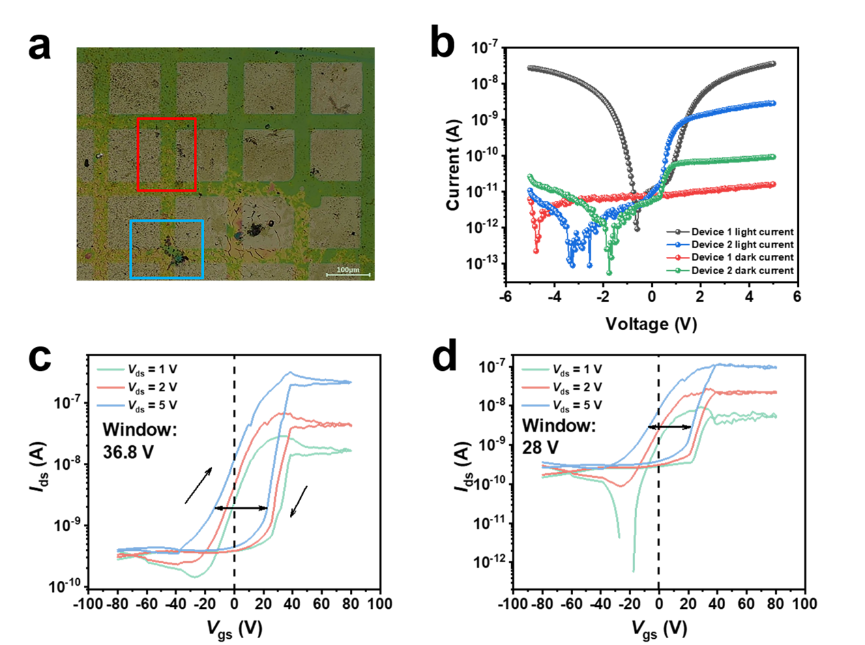


**Fig. S23 a,** Optical micrograph of the as-grown In₂Se₃ films (the red box exhibits superior crystallinity, while the film with holes in the blue box shows inferior crystallinity). **b,** Photocurrent and dark current at different crystallinity of the Ga₂O₃/In₂Se₃ film. **c,** Transfer characteristic curves in the superior crystallinity regions of the Ga₂O₃/In₂Se₃ film. **d,** Transfer characteristic curves in the inferior crystallinity regions of the Ga₂O₃/In₂Se₃ film


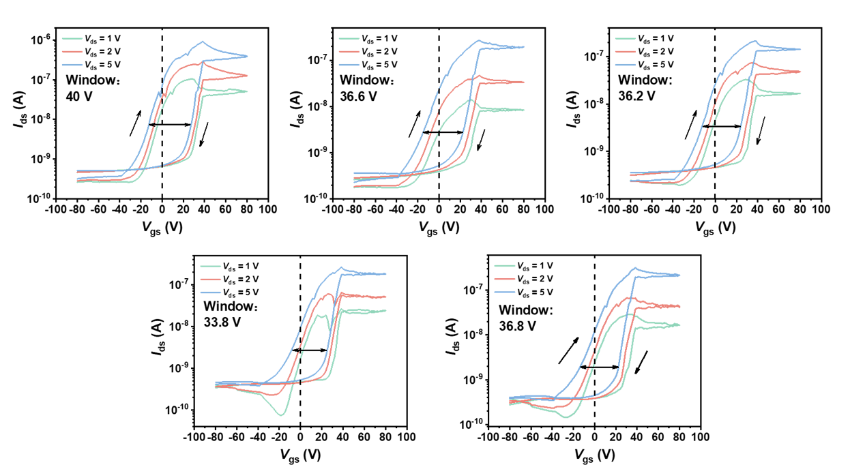


**Fig. S24** Field-effect transistor and hysteresis window at five distinct regions on the Ga₂O₃/In₂Se₃ film


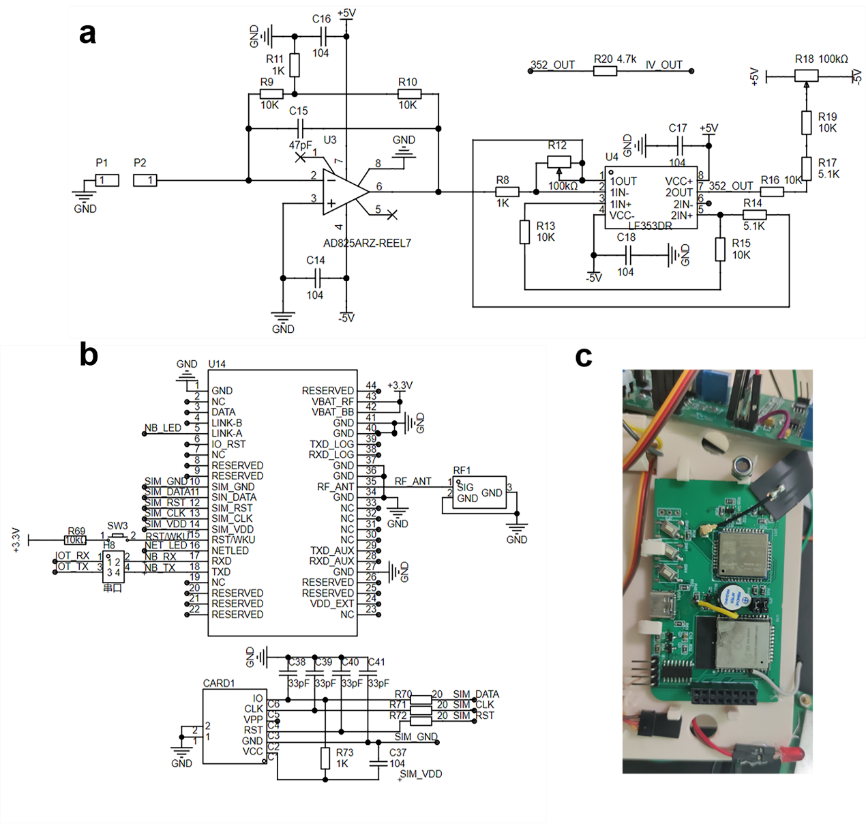


**Fig. S25 a,** Schematic diagram of the signal processing circuit. **b,** Schematic diagram of the NB_IOT Communication Module. **c,** Physical diagram of the flame detection circuits


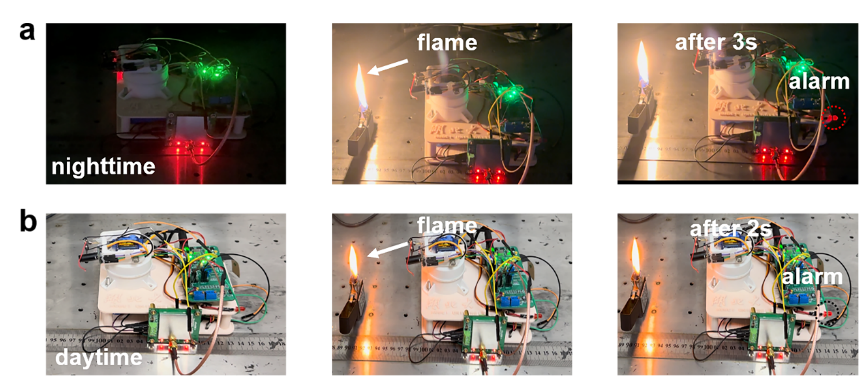


**Fig. S26 Flame detector system based on Fe-OES. Flame alarm system in nighttime conditions. a,** Flame alarm system. **b,** Flame ignition. **c,** Alarm of flame alarm system. Flame alarm system in daytime conditions. **d,** Flame alarm system. **e,** Flame ignition. **f,** Alarm of flame alarm system


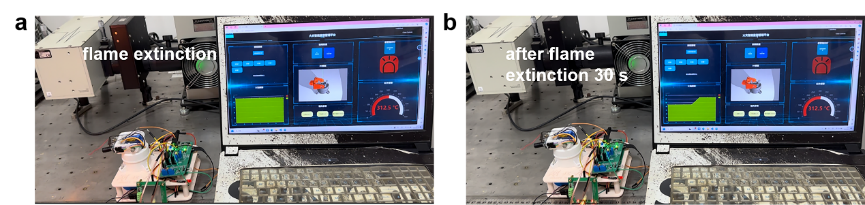


**Fig. S27 a,** Flame detection system after the flame is extinguished. **b,** Flame detection system after the flame has been extinguished for 30 s


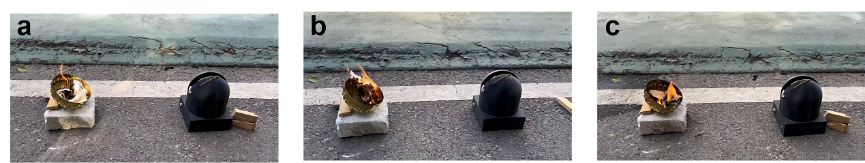


**Fig. S28** Real-world flame tests conducted in an outdoor sunny environment. **a,** Tissue paper combustion. **b,** Cotton combustion. **c,** Branches and leaves combustion


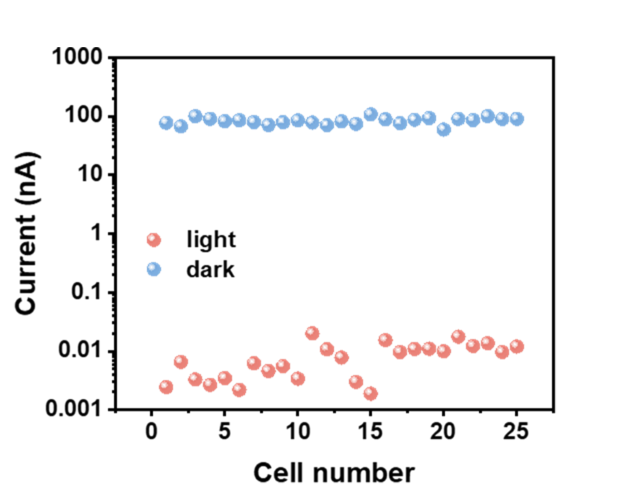


**Fig. S29** Distribution of photocurrent and dark current across the 5×5 Ga₂O₃/In₂Se₃ heterojunction array under 255 nm illumination (300 μW/cm²)


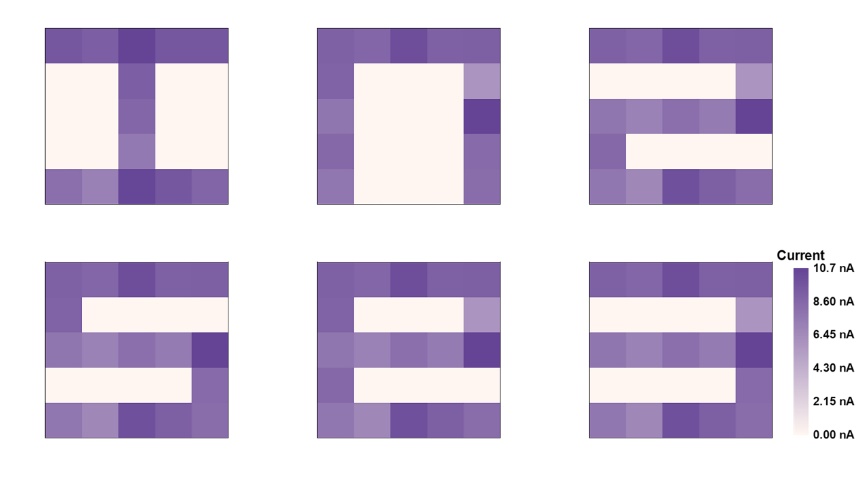


**Fig. S30** Two-dimensional photocurrent mapping of the 5×5 Ga₂O₃/In₂Se₃ heterojunction array under 255 nm illumination (300 μW/cm²)


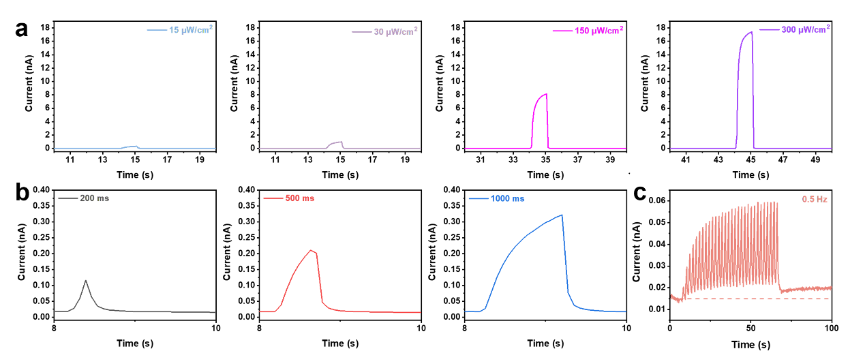


**Fig. S31 a,** PSC when optical pulses (15, 30, 150, and 300 μW/cm^2^, 500 ms pulses) were applied to the device, exhibiting higher photocurrent with increased light intensity. **b,** PSC when optical pulses with increased illumination time (0.2, 0.5, and 1 s, 15 μW/cm2 pulses) were applied to the device, a more prominent slow response caused by slow traps is observed. **c,** Excitatory PSC induced by optical pulses (15 μW/cm², 1000 ms duration, 0.5 Hz frequency) under a 1 V bias


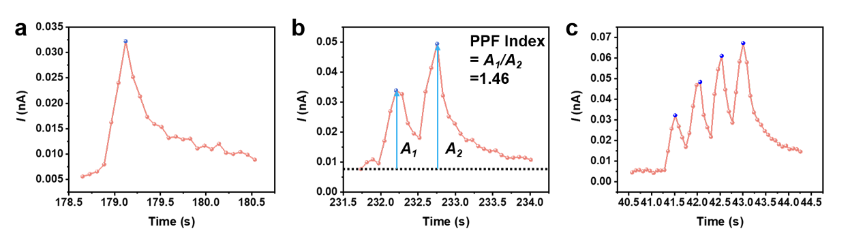


**Fig. S32 a,** Photoelectric synapse under a 200 ms single light pulse stimulation. **b,** Light response curves of two consecutive 200 ms light pulses, with an interval of 200 ms between the two consecutive light pulses. **c,** Response currents for photoelectric synapse under a 200 ms four consecutive light pulse stimulations


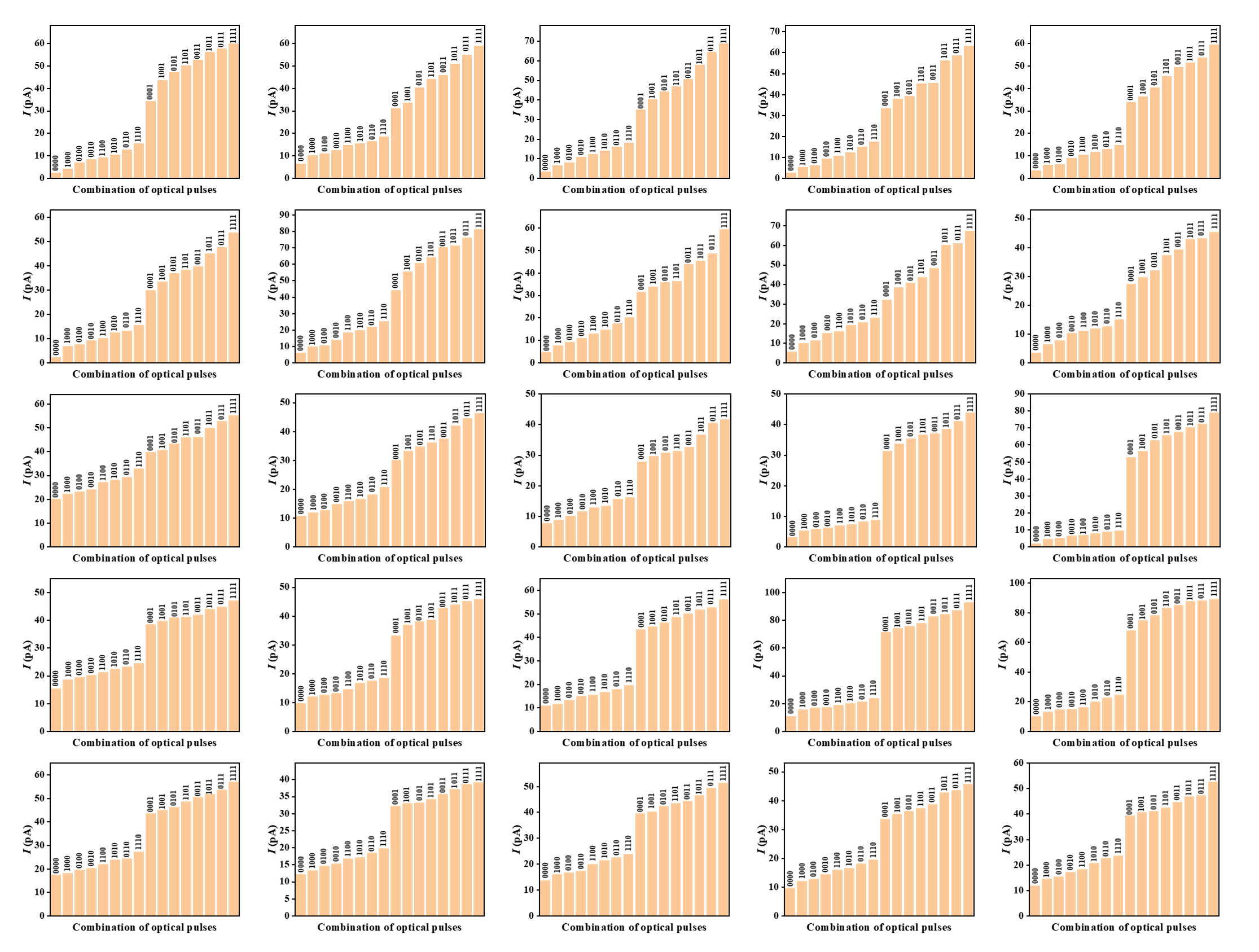


**Fig. S33** Response of the 5×5 Ga₂O₃/In₂Se₃ heterojunction array to a four-pulse optical stimulus


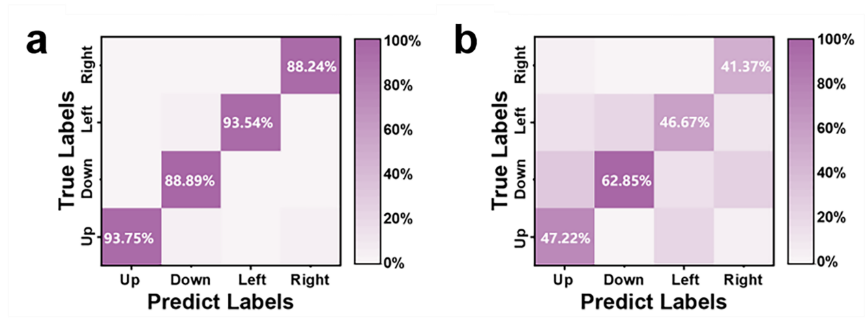


**Fig. S34** **a,** Confusion Matrix of Ga_2_O_3_/In_2_Se_3_-based sensors. **b,** Confusion Matrix of Ga_2_O_3_-based sensors


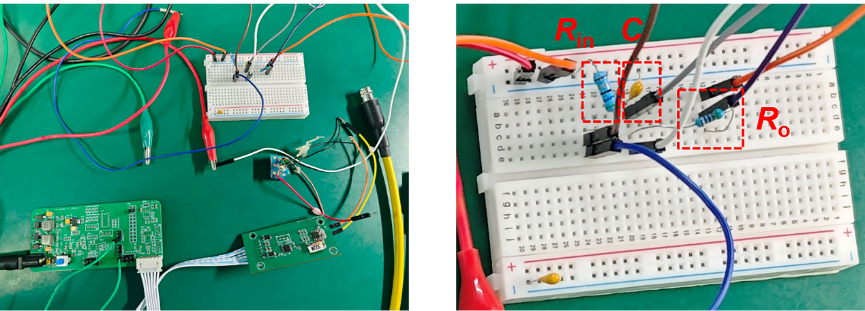


**Fig. S35** Physical hardware implementation of the leaky integrate-and-fire (LIF) neuron


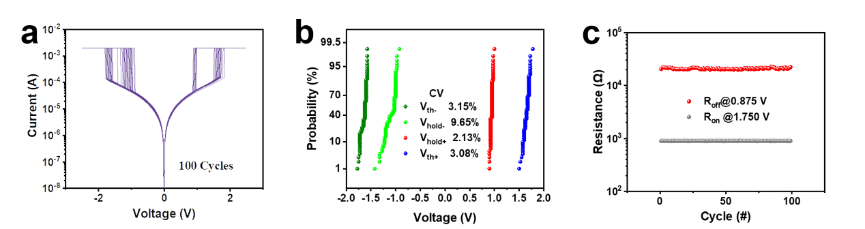


**Fig. S36 a**, 100 consecutive sweep cycles of the NbOₓ threshold switching device. **b**, Coefficient of variation (CV) of the switching parameters. **c**, Distribution of switching resistance over 100 operational cycles


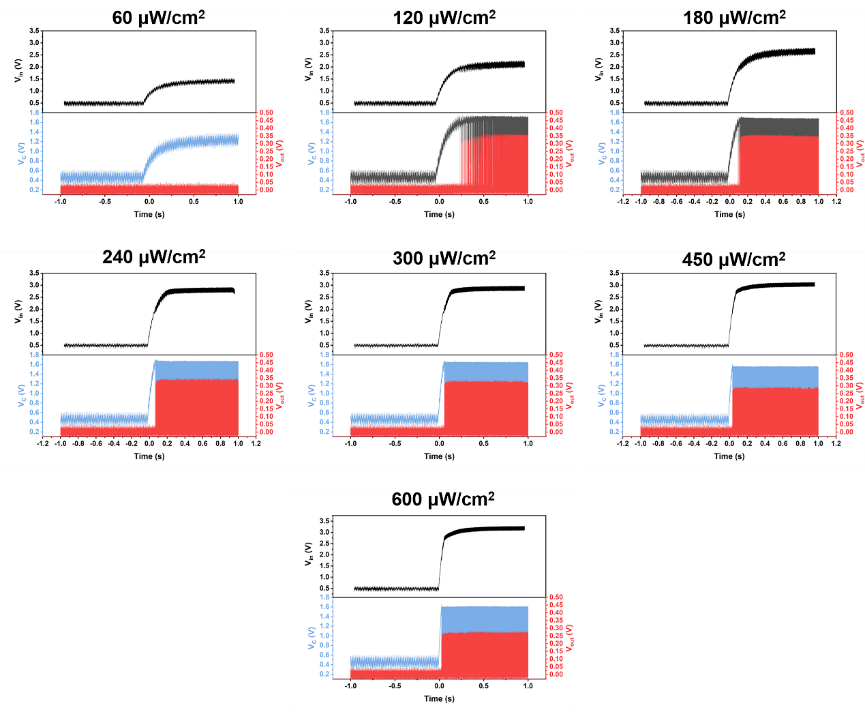


**Fig. S37** Schematic of input voltage, capacitance voltage and output voltage at different light intensities


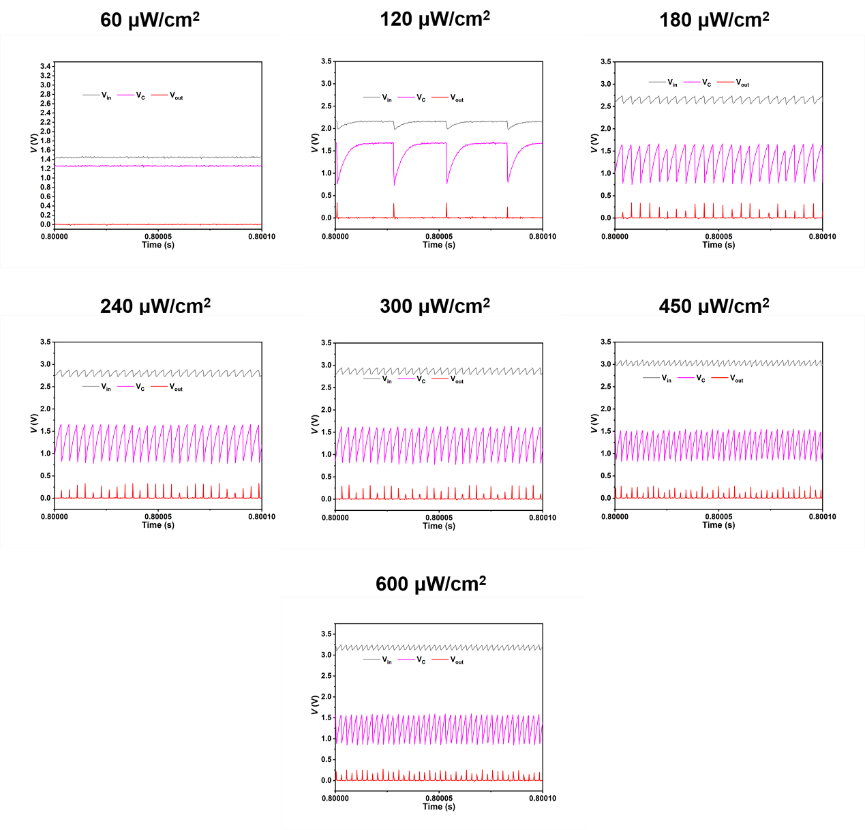


**Fig. S38** Schematic of input voltage, capacitor voltage and output voltage details at different light intensities


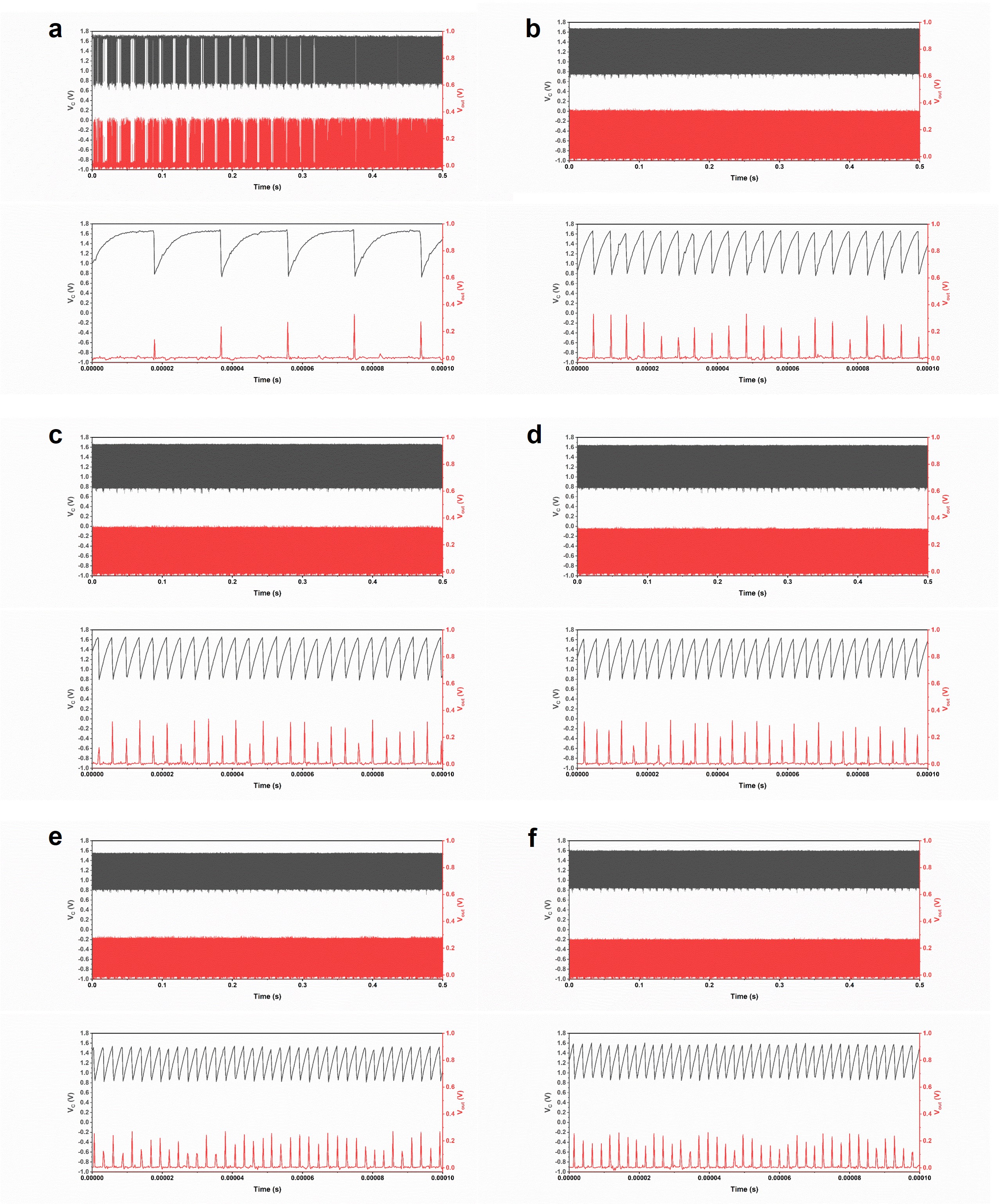


**Fig. S39** Long-term stability of VC and Vout of the LIF neuron under different optical intensities (upper: full-range view; lower: magnified view). **a,** 120 μW/cm². **b,** 180 μW/cm². **c,** 240 μW/cm². **d,** 300 μW/cm². **e,** 450 μW/cm². **f,** 600 μW/cm²

**Table 1** The specific effects of annealing treatments on Ga₂O₃ photodetectors

| **Mechanism** | **Specific effect** | **Refs.** |
| --- | --- | --- |
| Enhancement of crystalline quality | Reduction of dark current and enhancement of response speed | [S1] |
| Modulation of oxygen vacancies | Enhancement of responsivity and response speed | [S2] |
| Improvement of crystalline quality and reduction of oxygen vacancy defects | Enhancement of response speed with reduction of photocurrent and responsivity | [S3] |
| Significant suppression of oxygen vacancies | Reduction in responsivity and response speed | [S4] |
| Reduction of oxygen vacancy concentration | Reduction of dark current and enhancement of response speed | [S5] |

**Mechanisms of annealing**

The trade-off between responsivity and response speed, known as the "Responsivity-Speed Dilemma" [S1], is particularly pronounced in wide-bandgap semiconductors. Ga₂O₃ photodetectors exhibit persistent photoconductivity (PPC) effect, a phenomenon where the conductivity of the detector persists long after the cessation of illumination. This phenomenon is primarily attributed to deep-level defects, such as oxygen vacancies (V_O_), which trap photo-generated carriers can enhance non-radiative recombination and prolong the conductive state [S2, S3]. Furthermore, these defects contribute to higher dark current and inferior photodetection performance (e.g., low specific detectivity). Conversely, V_O_ can also temporarily trap electrons under illumination, influencing the photocurrent generation and consequently degrading the response speed [S4].

Therefore, modifying the intrinsic crystalline defects in Ga₂O₃ is crucial for enhancing device performance. Annealing engineering serves as a common strategy for this purpose. The specific effects of annealing treatments on Ga₂O₃ photodetectors, as reported in the literature, are summarized in Table 1. High-temperature annealing can effectively suppress intrinsic defects in Ga₂O₃, leading to devices with lower dark current and higher responsivity. This explains the significant performance improvement observed in the device annealed at 400°C. However, although annealing at even higher temperatures (e.g., 700 °C) leads to more pronounced defect suppression, it also results in a substantial reduction in photocurrent. This is because V_O_, while suppressed, can still act as temporary electron traps under illumination, adversely affecting the overall photoelectronic performance. This mechanism accounts for the performance degradation observed in the sample annealed at 700°C compared to the one annealed at 400 °C.

**Table 2** The comparison of responsivity and detectivity between Fe-OES and other reports

| **Device** | **I_dark_ (A)** | **PDCR** | **R (A/W)** | **D* (Jones)** | **LDR (db)** | **EQE (%)** | **Refs.** |
| --- | --- | --- | --- | --- | --- | --- | --- |
| GaO_X_  Ga_2_O_3_/MgO/Nb:STO  a-Ga_2_O_3_  Ga_2_O_3_/ZnO  TAPC/Ga_2_O_3_  IGZO-Ga_2_O_3_  PCDTBT/Ga_2_O_3_  Ga_2_O_3_/BFO  GaSe/*β*-Ga_2_O_3_  *α*-Ga_2_O_3_/polyaniline  *α*-Ga_2_O_3_  Ga_2_O_3_  *ε*-Ga_2_O_3_  In_2_Se_3_/*α*-Ga_2_O_3_ | 4.6×10^-14^(-10V)  2.2×10^-12^  3×10^-13^ (5V)  2.17×10^-9^  2×10^-14^ (0V)  1×10^-10^ (10V)  4.8×10^-13^  3.78×10^-12^  -  2.1×10^-13^  -  2×10^-13^ (15V)  3.34×10^-9^ (20V)  4.74×10^-13^ (1V) | 3×10^8^  -  3.9×10^7^  -  5.9×10^5^  -  -  1×10^5^  7.65×10^4^  -  -  1×10^6^  2.22×10^7^  2.63×10^5^ | 66.7  4.46×10^4^  733  2.49  1.4×10^-3^  284  187  0.012  52  8.2×10^-3^  1.65×10^4^  1.3  1.57×10^4^  118473 | 8×10^15^  5×10^16^  3.9×10^16^  1.98×10^14^  1.02×10^13^  5.06×10^14^  1.3×10^16^  6.1×10^12^  2.52×10^14^  6.63×10^13^  2.66×10^16^  1.46×10^14^  1.65×10^16^  4.91×10^17^ | -  81.9  -  -  -  -  -  -  -  -  -  -  -  100 | -  -  -  -  -  -  3.1×10^4^  0.34  2.54×10^4^  38.4  -  -  -  5.77×10^7^ | [S3]  [S6]  [S7]  [S8]  [S9]  [S10]  [S11]  [S12]  [S13]  [S14]  [S15]  [S16]  [S17]  This work |

**Table 3** Alarm response times for combustion tests of different materials

| Material | Alarm time (s) | | | | |
| --- | --- | --- | --- | --- | --- |
| Tissue paper  Cotton  Branches/leaves | 6  20  3 | 6  13  3 | 11  5  4 | 24  6  3 | 20  4  5 |

**Supplementary References**

1. X. Ma, Y. Zhang, P. Tan, X. Feng, Y. Hao et al., Alleviating trade-off between responsivity and response speed of Ga_2_O_3_ solar-blind photodetector *via* modulation of carrier redistribution and extraction accessibility. InfoMat **7**(9), e70016 (2025). <https://doi.org/10.1002/inf2.70016>
2. L. Luo, H. Huang, L. Yang, R. Hao, X. Hu et al., Ultra-fast gallium oxide solar-blind photodetector with novel thermal pulse treatment. Adv. Mater. **37**(12), 2414130 (2025). <https://doi.org/10.1002/adma.202414130>
3. X. Hou, X. Zhao, Y. Zhang, Z. Zhang, Y. Liu et al., High-performance harsh-environment-resistant GaOX solar-blind photodetectors *via* defect and doping engineering. Adv. Mater. **34**(1), 2106923 (2022). <https://doi.org/10.1002/adma.202106923>
4. T. Sheng, X.-Z. Liu, L.-X. Qian, B. Xu, Y.-Y. Zhang, Photoelectric properties of β-Ga_2_O_3_ thin films annealed at different conditions. Rare Met. **41**(4), 1375–1379 (2022). <https://doi.org/10.1007/s12598-015-0575-5>
5. C. Zhou, K. Liu, X. Chen, J. Feng, J. Yang et al., Performance improvement of amorphous Ga_2_O_3_ ultraviolet photodetector by annealing under oxygen atmosphere. J. Alloys Compd. **840**, 155585 (2020). <https://doi.org/10.1016/j.jallcom.2020.155585>
6. Q. Zhang, N. Li, T. Zhang, D. Dong, Y. Yang et al., Enhanced gain and detectivity of unipolar barrier solar blind avalanche photodetector *via* lattice and band engineering. Nat. Commun. **14**(1), 418 (2023). <https://doi.org/10.1038/s41467-023-36117-8>
7. Y. Qin, L.-H. Li, Z. Yu, F. Wu, D. Dong et al., Ultra-high performance amorphous Ga_2_O_3_ photodetector arrays for solar-blind imaging. Adv. Sci. **8**(20), 2101106 (2021). <https://doi.org/10.1002/advs.202101106>
8. H. Wang, J. Ma, H. Chen, L. Wang, P. Li et al., Ferroelectricity enhanced self-powered solar-blind UV photodetector based on Ga_2_O_3_/ZnO: V heterojunction. Mater. Today Phys. **30**, 100929 (2023). <https://doi.org/10.1016/j.mtphys.2022.100929>
9. C. Wu, F. Wu, C. Ma, S. Li, A. Liu et al., A general strategy to ultrasensitive Ga_2_O_3_ based self-powered solar-blind photodetectors. Mater. Today Phys. **23**, 100643 (2022). <https://doi.org/10.1016/j.mtphys.2022.100643>
10. S. Zhou, H. Zhang, X. Peng, H. Liu, H. Li et al., Fully transparent and high-performance ε-Ga_2_O_3_ photodetector arrays for solar-blind imaging and deep-ultraviolet communication. Adv. Photonics Res. **3**(11), 2200192 (2022). <https://doi.org/10.1002/adpr.202200192>
11. Y. Wang, Z. Lin, J. Ma, Y. Wu, H. Yuan et al., Multifunctional solar-blind ultraviolet photodetectors based on p-PCDTBT/n-Ga_2_O_3_ heterojunction with high photoresponse. InfoMat **6**(2), e12503 (2024). <https://doi.org/10.1002/inf2.12503>
12. G. Ma, W. Jiang, W. Sun, Z. Yan, B. Sun et al., A broadband UV-visible photodetector based on a Ga_2_O_3_/BFO heterojunction. Phys. Scr. **96**(12), 125823 (2021). <https://doi.org/10.1088/1402-4896/ac2758>
13. Y. Wang, Y. Tang, H. Li, Z. Yang, Q. Zhang et al., P-GaSe/n-Ga_2_O_3_ van der Waals heterostructure photodetector at solar-blind wavelengths with ultrahigh responsivity and detectivity. ACS Photonics **8**(8), 2256–2264 (2021). <https://doi.org/10.1021/acsphotonics.1c00015>
14. X.Y. Sun, X.H. Chen, J.G. Hao, Z.P. Wang, Y. Xu et al., A self-powered solar-blind photodetector based on polyaniline/*α*-Ga_2_O_3_ p–n heterojunction. Appl. Phys. Lett. **119**(14), 141601 (2021). <https://doi.org/10.1063/5.0059061>
15. Q. Zhang, D. Dong, T. Zhang, T. Zhou, Y. Yang et al., Over 5 × 103-fold enhancement of responsivity in Ga_2_O_3_-based solar blind photodetector *via* acousto–photoelectric coupling. ACS Nano **17**(23), 24033–24041 (2023). <https://doi.org/10.1021/acsnano.3c08938>
16. M. Ding, K. Liang, S. Yu, X. Zhao, H. Ren et al., Aqueous-printed Ga_2_O_3_ films for high-performance flexible and heat-resistant deep ultraviolet photodetector and array. Adv. Opt. Mater. **10**(16), 2200512 (2022). <https://doi.org/10.1002/adom.202200512>
17. W. Zhang, W. Wang, J. Wei, S. Xia, J. Zhang et al., Photocarrier transport reconstruction and dramatical performance enhancement in ultrawide-bandgap *ε*-Ga_2_O_3_ photodetectors via surface defect passivation. Mater. Today Phys. **38,** 101280–101280 (2023). <https://doi.org/10.1016/j.mtphys.2023.101280>

**Appendix**

**Code for CNN-motion flame**

import os

import numpy as np

import tensorflow as tf

from tensorflow.keras import layers, models

import matplotlib.pyplot as plt

from sklearn.model_selection import train_test_split

from sklearn.metrics import confusion_matrix, classification_report

import pandas as pd

import time

import seaborn as sns

data_dir = r'E: \database_Tra_test'

def load_data(data_dir):

categories = ['up', 'down', 'left', 'right']

images = []

labels = []

for label, category in enumerate(categories):

category_path = os.path.join(data_dir, category)

for file in os.listdir(category_path):

img_path = os.path.join(category_path, file)

img = tf.keras.preprocessing.image.load_img(img_path, color_mode='grayscale', target_size=(50, 50))

img_array = tf.keras.preprocessing.image.img_to_array(img)

images.append(img_array)

labels.append(label)

images = np.array(images).reshape(-1, 50, 50, 1)

labels = np.array(labels)

return images, labels

def get_flops(model):

session = tf.compat.v1.Session()

graph = tf.compat.v1.get_default_graph()

with graph.as_default():

with session.as_default():

run_meta = tf.compat.v1.RunMetadata()

opts = tf.compat.v1.profiler.ProfileOptionBuilder.float_operation()

flops = tf.compat.v1.profiler.profile(

graph=graph,

run_meta=run_meta,

cmd='op',

options=opts)

return flops.total_float_ops

def analyze_energy_consumption(model, X_sample, training_time, num_epochs, num_train_samples):

try:

total_flops = get_flops(model)

print(f"ModleFLOPs: {total_flops:.2e}")

inference_flops_per_sample = total_flops

hardware_efficiency = xxx # hardware parameter

training_energy = (total_flops * num_epochs * num_train_samples) / hardware_efficiency

inference_energy_per_sample = inference_flops_per_sample / hardware_efficiency

except Exception as e:

print(f"FLOPs Calculation failed: {e}")

total_flops = None

training_energy = None

inference_energy_per_sample = None

avg_power = 50 # Watts

training_energy_time_based = avg_power * training_time # Joules

start_time = time.time()

model.predict(X_sample[np.newaxis,...])

inference_time = time.time() - start_time

inference_energy_time_based = avg_power * inference_time

return {

"flops": total_flops,

"training_energy_flops": training_energy,

"training_energy_time": training_energy_time_based,

"inference_time": inference_time,

"inference_energy": inference_energy_time_based

}

def plot_and_save_confusion_matrix(y_true, y_pred, classes, save_path):

cm = confusion_matrix(y_true, y_pred)

cm_df = pd.DataFrame(cm, index=classes, columns=classes)

cm_excel_path = os.path.join(save_path, 'confusion_matrix.xlsx')

cm_df.to_excel(cm_excel_path)

print(f" The confusion matrix has been saved to: {cm_excel_path}")

report = classification_report(y_true, y_pred, target_names=classes, output_dict=True)

report_df = pd.DataFrame(report).transpose()

report_path = os.path.join(save_path, 'classification_report.xlsx')

report_df.to_excel(report_path)

print(f" The classification report has been saved to: {report_path}")

plt.**Fig.**(figsize=(10, 8))

sns.heatmap(cm, annot=True, fmt='d', cmap='Blues',

xticklabels=classes, yticklabels=classes)

plt.title('Confusion Matrix')

plt.ylabel('True Label')

plt.xlabel('Predicted Label')

cm_image_path = os.path.join(save_path, 'confusion_matrix.png')

plt.savefig(cm_image_path, dpi=300, bbox_inches='tight')

print(f" The confusion matrix image has been saved to: {cm_image_path}")

plt.show()

return cm_df, report_df

images, labels = load_data(data_dir)

X_train, X_test, y_train, y_test = train_test_split(images, labels, test_size=0.3, random_state=4)

model = models.Sequential([

layers.Conv2D(8, (2, 2), activation='relu', input_shape=(50, 50, 1)),

layers.MaxPooling2D((2, 2)),

layers.Flatten(),

layers.Dense(64, activation='relu'),

layers.Dense(4, activation='softmax')

])

model.compile(optimizer='adam', loss='sparse_categorical_crossentropy', metrics=['accuracy'])

model.summary()

start_time = time.time()

history = model.fit(X_train, y_train, epochs=20, validation_data=(X_test, y_test))

training_time = time.time() - start_time

print(f" Training time: {training_time:.2f} seconds")

energy_results = analyze_energy_consumption(

model,

X_test[0],

training_time,

num_epochs=20,

num_train_samples=len(X_train)

)

plt.**Fig.**(figsize=(12, 4))

plt.subplot(1, 2, 1)

plt.plot(history.history['loss'], label='Training Loss')

plt.plot(history.history['val_loss'], label='Validation Loss')

plt.legend()

plt.title('Loss')

plt.subplot(1, 2, 2)

plt.plot(history.history['accuracy'], label='Training Accuracy')

plt.plot(history.history['val_accuracy'], label='Validation Accuracy')

plt.legend()

plt.title('Accuracy')

plt.show()

model.save('E:\\model\\flame_direction_model_conventional.h5')

test_loss, test_acc = model.evaluate(X_test, y_test)

print(f'Test accuracy: {test_acc}')

y_pred = model.predict(X_test)

y_pred_classes = np.argmax(y_pred, axis=1)

class_names = ['up', 'down', 'left', 'right']

results_dir = r'E:\results'

os.makedirs(results_dir, exist_ok=True)

cm_df, report_df = plot_and_save_confusion_matrix(y_test, y_pred_classes, class_names, results_dir)

train_acc = history.history['accuracy']

val_acc = history.history['val_accuracy']

accuracy_data = pd.DataFrame({

'Epoch': range(1, len(train_acc) + 1),

'Training Accuracy': train_acc,

'Validation Accuracy': val_acc

})

energy_data = pd.DataFrame({

'Metric': [

'Training Time',

'Training Energy (Time-based)',

'Inference Time per sample',

'Inference Energy per sample (Time-based)'

],

'Value': [

training_time,

energy_results['training_energy_time'],

energy_results['inference_time'],

energy_results['inference_energy']

],

'Unit': ['Seconds', 'Joules', 'Seconds', 'Joules']

})

if energy_results['flops'] is not None:

flops_data = pd.DataFrame({

'Metric': ['Total FLOPs', 'Training Energy (FLOPs-based)'],

'Value': [energy_results['flops'], energy_results['training_energy_flops']],

'Unit': ['FLOPs', 'Joules']

})

energy_data = pd.concat([energy_data, flops_data], ignore_index=True)

output_path = os.path.join(results_dir, 'accuracy_data.xlsx')

accuracy_data.to_excel(output_path, index=False)

energy_output_path = os.path.join(results_dir, 'energy_analysis.xlsx')

energy_data.to_excel(energy_output_path, index=False)

print(f'Accuracy data successfully saved to {output_path}')

print(f'Energy analysis data successfully saved to {energy_output_path}')

print("\n=== Model Performance Summary ===")

print(f" Test accuracy: {test_acc:.4f}")

print(f" Training time: {training_time:.2f} seconds")

print(f" Single inference time: {energy_results['inference_time']:.6f} seconds")

print(f" Single inference energy consumption: {energy_results['inference_energy']:.6f} Joule")

**Code for simulation of SNN**

import time

import torch

import torchvision

import torchvision.transforms as transforms

import torch.utils.data

import torch.nn as nn

import torch.nn.functional as F

import torch.optim as optim

import numpy as np

import pandas as pd

from spikingjelly.activation_based import neuron, functional, layer

class CustomFrequencyEncoder(nn.Module):

def __init__(self, A1, t1, y0, T_steps):

super(CustomFrequencyEncoder, self).__init__()

self.A1 = A1

self.t1 = t1

self.y0 = y0

self.T_steps = T_steps

def encode(self, inputs):

inputs_normalized = inputs.view(-1)

frequency = self.A1 * torch.exp(-inputs_normalized / self.t1) + self.y0

frequency = torch.clamp(frequency, min=0.01, max=1.0)

frequency = frequency.view(inputs.shape)

spike_train = torch.rand_like(inputs).le(frequency.unsqueeze(0).expand(self.T_steps, *inputs.shape)).float()

return spike_train

transform = transforms.Compose([transforms.ToTensor()])

trainset = torchvision.datasets.MNIST(root='../mnist', train=True, download=False, transform=transform)

trainloader = torch.utils.data.DataLoader(trainset, batch_size=256, shuffle=True, num_workers=0)

testset = torchvision.datasets.MNIST(root='../mnist', train=False, download=False, transform=transform)

testloader = torch.utils.data.DataLoader(testset, batch_size=256, shuffle=False, num_workers=0)

net = nn.Sequential(

layer.Flatten(start_dim=1),

layer.Linear(28 * 28, 128, bias=False),

neuron.LIFNode(tau=2.0),

layer.Linear(128, 10, bias=False),

neuron.LIFNode(tau=2.0)

)

functional.set_step_mode(net, step_mode='m')

criterion = nn.MSELoss()

optimizer = optim.Adam(net.parameters())

encoder = CustomFrequencyEncoder(A1=-0.53087, t1=0.73155, y0=0.52776, T_steps=50)

epoch_loss = []

train_acc = []

test_acc = []

all_spikes = []

all_labels = []

start = time.time()

for epoch in range(30):

running_loss = 0.0

train_correct = 0

train_total = 0

for i, data in enumerate(trainloader, 0):

inputs, labels = data

labels_onehot = F.one_hot(labels, 10).float()

inputs_spks_Time = encoder.encode(inputs)

optimizer.zero_grad()

outputs_spks_Time = net(inputs_spks_Time)

for t in range(50):

if t == 0:

out_spikes_counter = outputs_spks_Time[0]

else:

out_spikes_counter += outputs_spks_Time[t]

out_spikes_counter_frequency = out_spikes_counter / 50

loss = criterion(out_spikes_counter_frequency, labels_onehot)

loss.backward()

optimizer.step()

functional.reset_net(net)

running_loss += loss.item()

train_correct += (out_spikes_counter_frequency.argmax(-1) == labels).sum().item()

train_total += labels.size(0)

epoch_loss.append(running_loss / len(trainloader))

train_acc.append(train_correct / train_total)

net.eval()

test_correct = 0

test_total = 0

with torch.no_grad():

for inputs, labels in testloader:

labels_onehot = F.one_hot(labels, 10).float()

inputs_spks_Time = encoder.encode(inputs)

outputs_spks_Time = net(inputs_spks_Time)

out_spikes_counter = sum(outputs_spks_Time[t] for t in range(50))

out_spikes_counter_frequency = (out_spikes_counter / 50).cpu().numpy()

test_correct += (out_spikes_counter_frequency.argmax(-1) == labels.numpy()).sum()

test_total += labels.size(0)

all_spikes.append(out_spikes_counter_frequency)

all_labels.append(labels.cpu().numpy())

functional.reset_net(net)

test_acc.append(test_correct / test_total)

net.train()

print(f'Epoch [{epoch+1}/30], Loss: {epoch_loss[-1]:.4f}, Train Acc: {train_acc[-1]:.4f}, Test Acc: {test_acc[-1]:.4f}')

label_spikes_mean = np.zeros((10, 10))

label_counts = np.zeros(10)

for i in range(len(all_labels)):

for label, spikes in zip(all_labels[i], all_spikes[i]):

label_spikes_mean[label] += spikes

label_counts[label] += 1

label_spikes_mean /= label_counts[:, None]

label_spikes_df = pd.DataFrame(label_spikes_mean, columns=[f"Neuron {i}" for i in range(10)])

label_spikes_df.index = [f"Label {i}" for i in range(10)]

label_spikes_df.to_csv("label_spikes_mean.csv", index=True)

print("Saved: label_spikes_mean.csv")

flat_spikes = np.concatenate(all_spikes, axis=0)

flat_labels = np.concatenate(all_labels, axis=0)

spikes_df = pd.DataFrame(flat_spikes, columns=[f"Neuron {i}" for i in range(10)])

spikes_df["Label"] = flat_labels

spikes_df.to_csv("spike_results.csv", index=False)

print("Saved: spike_results.csv")

accuracy_df = pd.DataFrame({

"Epoch": list(range(1, 31)),

"Train Accuracy": train_acc,

"Test Accuracy": test_acc

})

accuracy_df.to_csv("accuracy_results.csv", index=False)

print("Saved: accuracy_results.csv")
